# Dissecting the phyloepidemiology of *Trypanosoma cruzi* I (TcI) in Brazil by the use of high resolution genetic markers

**DOI:** 10.1371/journal.pntd.0006466

**Published:** 2018-05-21

**Authors:** Fabiola Roman, Samanta das Chagas Xavier, Louisa A. Messenger, Márcio G. Pavan, Michael A. Miles, Ana María Jansen, Matthew Yeo

**Affiliations:** 1 Laboratório de Bleiologia de Tripanossomatídeos, Instituto Oswaldo Cruz, Fundação Oswaldo Cruz, Rio de Janeiro, Rio de Janeiro, Brasil; 2 Faculty of Infectious and Tropical Diseases, Department of Pathogen Molecular Biology, London School of Hygiene and Tropical Medicine, London, United Kingdom; 3 Laboratório de Mosquitos Transmissores de Hematozoários, Instituto Oswaldo Cruz, Fundação Oswaldo Cruz, Rio de Janeiro, Rio de Janeiro, Brazil; Temple University, UNITED STATES

## Abstract

**Background:**

*Trypanosoma cruzi*, the causal agent of Chagas disease, is monophyletic but genetically heterogeneous. It is currently represented by six genetic lineages (Discrete Typing Units, DTUs) designated TcI-TcVI. TcI is the most geographically widespread and genetically heterogeneous lineage, this as is evidenced by a wide range of genetic markers applied to isolates spanning a vast geographic range in Latin America.

**Methodology/Principal findings:**

In total, 78 TcI isolated from hosts and vectors distributed in 5 different biomes of Brazil, were analyzed using 6 nuclear housekeeping genes, 25 microsatellite loci and one mitochondrial marker. Nuclear markers reveal substantial genetic diversity, significant gene flow between biomes, incongruence in phylogenies, and haplotypic analysis indicative of intra-DTU genetic exchange. Phylogenetic reconstructions based on mitochondrial and nuclear loci were incongruent, and consistent with introgression. Structure analysis of microsatellite data reveals that, amongst biomes, the Amazon is the most genetically diverse and experiences the lowest level of gene flow. Investigation of population structure based on the host species/genus, indicated that *Didelphis marsupialis* might play a role as the main disperser of TcI.

**Conclusions/Significance:**

The present work considers a large TcI sample from different hosts and vectors spanning multiple ecologically diverse biomes in Brazil. Importantly, we combine fast and slow evolving markers to contribute to the epizootiological understanding of TcI in five distinct Brazilian biomes. This constitutes the first instance in which MLST analysis was combined with the use of MLMT and maxicircle markers to evaluate the genetic diversity of TcI isolates in Brazil. Our results demonstrate the existence of substantial genetic diversity and the occurrence of introgression events. We provide evidence of genetic exchange in TcI isolates from Brazil and of the relative isolation of TcI in the Amazon biome. We observe the absence of strict associations with TcI genotypes to geographic areas and/or host species.

## Author summary

*T*. *cruzi* is a zoonotic protozoan parasite infecting mammals and widely dispersed throughout endemic Latin America. It is known to possess considerable genetic diversity, comprising six discrete genetic lineages designated Discrete Typing Units (DTUs) TcI-TcVI. TcI is the most genetically diverse DTU and the most frequently sampled lineage in Brazil. We use a combination of high resolution molecular techniques to analyze the genetic diversity of Brazilian TcI isolates obtained from a wide geographical area encompassing five distinct biomes isolated from different mammal hosts and insect vectors. The results reveal significant genetic diversity and no clear association of genotypes with areas or host/vector species. Evidence from incongruent phylogenetic topologies based on nuclear and mitochondrial markers are indicative of genetic exchange and/or introgression events. The relevance of these findings in the context of population structure, ecology and epizootiology is discussed.

## Introduction

*Trypanosoma cruzi*, a protozoan parasite (Kinetoplastidea: Trypanosomatidae), is known to possess a complex epidemiology and is widely distributed from the southern states of the United States of America to the Argentinian Patagonia. *T*. *cruzi* is a pervasive zoonosis capable of affecting more than 150 domestic and wild mammal species, distributed across 8 orders. *T*. *cruzi* infection in humans, may result in Chagas disease [1–3].Transmission to humans is mainly vectorial in endemic areas and over 100 species of hematophagous triatomine insects can harbor the parasites [4, 5]. Moreover, migration of individuals from highly endemic regions to the United States and Europe has resulted in significant public health concerns in recipient countries [6]. Domestic transmission of Chagas disease (CD) in Brazil by *Triatoma infestans* has been successfully interrupted [7]. However, human infection by *T*. *cruzi* is re-emerging as a food-borne disease in previously non-endemic areas [8–10]. Annual outbreaks have occurred, particulary in the northern Brazilian Amazon region during the past decade. Here, some local products derived from fruit juice have been contaminated with infected feces of triatomine bugs of different genera [9, 11, 12].

*T*. *cruzi* is characterized by a remarkable genetic heterogeneity [13, 14] and is currently comprised of six lineages or discrete typing units (DTUs), designated TcI to TcVI [15, 16]. In addition, recent evidence also supports the existence of a seventh lineage (TcBat) associated to bats [17]. The most genetically distant DTUs are TcI and TcII [18]. The evolutionary origins of TcIII and TcIV was initially proposed to be the result of an old hybridization between TcI and TcII [19], however more recent evidence shows that TcIII and TcIV have no hybrid origin, but rather are a monophyletic group with TcI that diverged from TcII [20, 21]. TcV and TcVI are known hybrid lineages which share haplotypes with TcII and TcIII [22, 23]. Whether given subpopulations of the parasite are associated with particular vector or host species or with distinct human disease characteristics is still unresolved.

TcI is the most frequently isolated DTU in the sylvatic environment, infecting diverse host and vector species across the Americas with an ancestral parental origin estimated at ~0.5–0.9 MYA [24, 23]. In Brazil, it is also the most widely distributed DTU, in terms of geography and diversity of host and vector species. Furthermore, it is, by far, the most genetically diverse DTU [25–30]. Llewellyn et al. [31] applied Multilocus Microsatellite Typing (MLMT) to the study of TcI population substructure in samples that originated from eight countries, isolated from 18 host and vector species, across 48 tandem repeats [32]. Results revealed extensive intra DTU diversity and spatial structuring of specific genotypes associated with acute oral outbreaks or vectorial infections in Venezuela. In addition, remarkable genetic diversity, through multiclonality, was observed when a single *Didelphis* reservoir host of TcI was studied [33].

Attempts to subdivide TcI strains into epidemiologically relevant groups are ongoing [34]. Herrera et al. [28] and Cura et al. [35] described five haplotypes associated with transmission cycles in Colombia, Chile and Bolivia. Ramirez et al. [36], used MLST to identify TcI genotypes specifically associated with human infection (TcI_DOM_) and others associated with peridomestic/sylvatic areas. MLST exploits nucleotide diversity present in four to ten single-copy housekeeping genes and has previously been applied to the study of *T*. *cruzi* using different marker combinations for lineage assignment and intraspecific characterization [18, 37]. Evidence for genetic exchange in TcI has been reported, for example, in strains isolated from *Didelphis marsupialis* and *Rhodnius prolixus* in the Amazon Basin [38] and in a domestic/peridomestic TcI population in Ecuador [39]. Experimental generation of intra-lineage hybrids suggest that TcI also displays a potential for genetic exchange [40].

Mitochondrial DNA in *T*. *cruzi* has a unique structure and function consisting of approximately 20–50 maxicircles (~20kb) and thousands of smaller minicircles (~1.4kb) [38]. Maxicircle DNA is uniparentally inherited and represents a useful taxonomic marker as it is highly mutable in comparison to nuclear DNA. Messenger et al. [41] developed a high resolution maxicircle multilocus sequence typing (mtMLST) scheme to describe intra-DTU diversity in TcI, revealing multiple mitochondrial introgression events and heteroplasmy within South American TcI. Introgression had already been detected in North America [21, 22] and in Brazil [42] and also Bolivia [43].

Together these studies illustrate several remarkable characteristics of TcI, namely the immense geographic distribution, diversity of host and vector species, extensive genetic diversity, and the capacity for genetic exchange. However, little is known about TcI in Brazil and extraordinarily there is only one relevant Brazil centric publication specifically addressing diversity of TcI [42]. Unlike Colombia and Venezuela, in Brazil there is no evidence of population substructure in the context of geographical distribution of intra DTU genotypes, distribution of host/vector species, or genotypes associated with acute outbreaks of CD in Brazil. In the present work, we comprehensively analyze a large cohort of Brazilian TcI isolates from five ecologically disparate biomes. Through the use of high resolution nuclear markers (MLST and MLMT) and a maxicircle region (*COII*), we investigate the phyloepizootiology of TcI from different Brazilian biomes. The study described herein was conducted with the following major hypothesis: DTU I of *T*. *cruzi* in Brazilian isolates displays extensive heterogeneity with no particular association of subpopulations to geographic areas, or host/vector species.

## Materials and methods

### Parasite isolates

A total of 78 TcI isolates were supplied by Coleção de *Trypanosoma* sp de Mamíferos Silvestres, Domésticos e Vetores COLTRYP/FIOCRUZ. deposited by several researchers and maintained in liquid nitrogen. DNA was extracted immediately following initial isolation in NNN medium and one round of expansion in LIT. The isolates had previously been confirmed as TcI using Mini-Exon PCR [44].

In this work, TcI isolates were characterized using three high resolution methods comprising MLST, MLMT and maxicircle sequencing (COII) using appropriate reference isolates. Full isolate details are shown in S1 Table and include characterization methods applied to each sample, isolate localities and collection dates. To increase the robustness of the results, microsatellite information from 50 additional isolates, published by Lima et al. [42], was included in our MLMT analyses.

**Fig 1:**
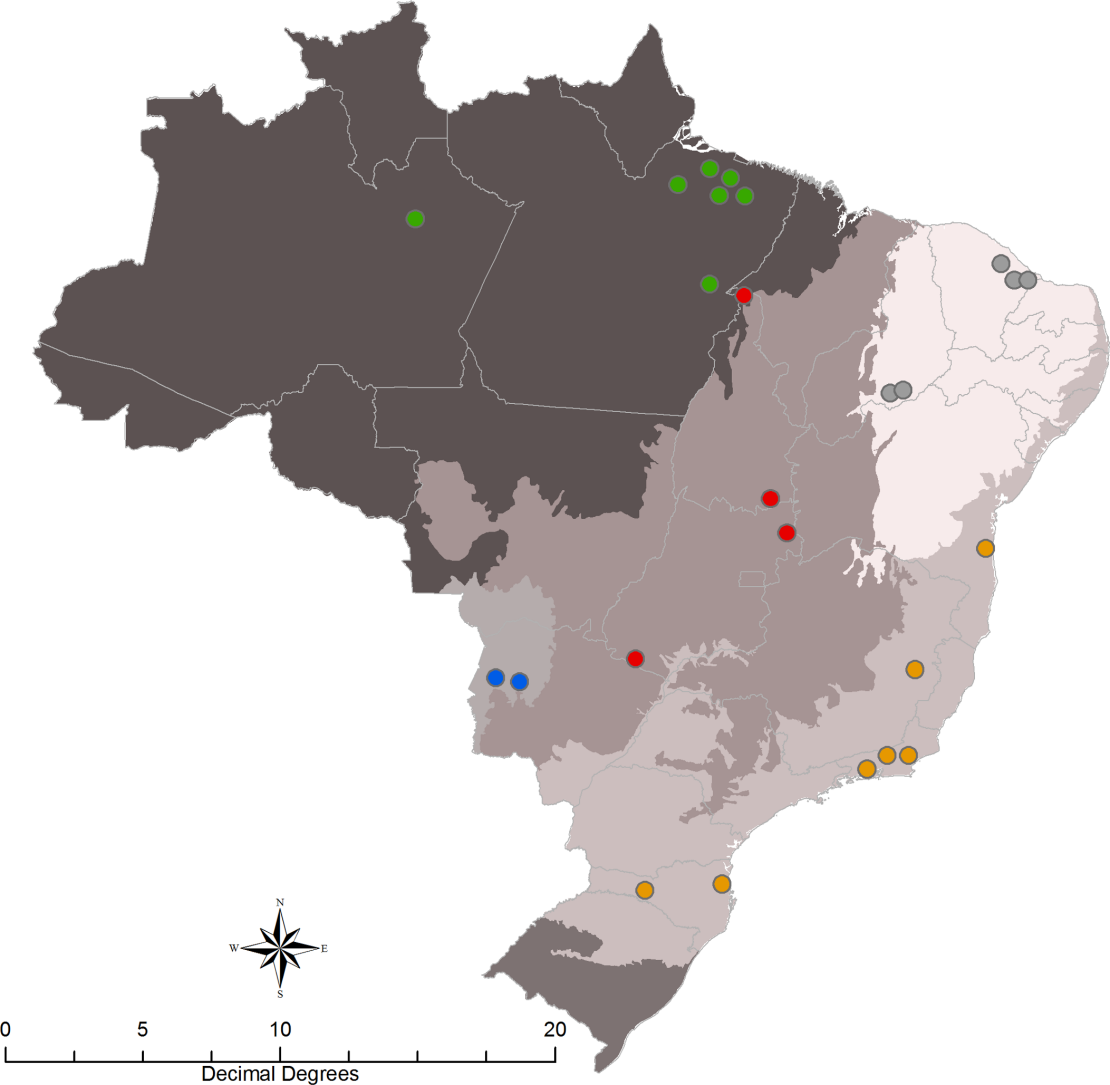
Map of the spatial distribution of TcI isolates used in the study. Colours represent the different biomes where samples were obtained: Green: Amazon; Orange: Atlantic Forest; Red: Cerrado. Blue: Pantanal. Grey: Caatinga.

Isolates originated from vector and mammalian reservoir hosts across five Brazilian biomes; namely Atlantic Forest, Amazon, Caatinga, Cerrado, and Pantanal (Fig 1 and S1 Table).

**The Cerrado** biome is primarily open scrubland (savannah) covering approximately 2 million km^2^ of Central Brazil, comprising 23% of the total land surface area [45]. Scrubland is interspersed with gallery forests and is seasonally dry but with permanent swamplands dominated by *Mauritia flexuosa* palm trees [46].

**The Pantanal** biome is a large seasonal floodplain covering approximately 140000 km^2^ at the core of South America [47]. It is a biodiversity hotspot and freshwater ecosystem of global significance containing diverse mammal species and habitats. Climate instability results in periodic floods and droughts, affecting the population number and behavior of some species [48].

The **Amazon** biome is in the largest hydrographic basin of the world, comprising 44% of the South American subcontinent. The biome is a complex mosaic of very diverse ecosystems, dominated by tropical rain forests, with semi-arid regions, and a variety of man-made landscapes. The Amazon biome contains the greatest biological diversity (in absolute terms) on the planet [49].

**The Atlantic Forest** biome extends from the south of Pernambuco to the south of Rio Grande do Sul, and it is characterized by humid tropical forest. This biome is extensively impacted by human activities. It originally encompassed 12 percent of the national territory but only 1 to 5 percent (less than 100,000 km^2^) is intact today [50]. Containing more than 8,000 endemic species, the Atlantic Forest is recognized as one of the world’s most significant biodiversity hotspots [51].

**The Caatinga** biome in northeast Brazil, comprises a semi-arid ecological landscape with only 1% of its territory currently conserved, it is threatened by agriculture and cattle ranching [52]. This biome is characterized by clay and sandy soils with open plains supporting flora that is typical of semi-arid regions [53].

### Multilocus sequence typing (MLST)

#### MLST Loci

Ten housekeeping gene fragments were initially considered with the objective of detecting intra-lineage variation within TcI. Fragments were selected from Yeo et al. [37] and Diosque et al. [18] using the following rationale. Firstly, those known to be phylogenetically informative for TcI based on typing efficiency and discriminatory power [18, 37]; secondly, reliable PCR amplification, and thirdly consistent sequencing quality in both 5’ and 3’ directions. Two genes previously described by Yeo et al. [37]: mitochondrial peroxidase (*TcMPX)* and RNA-binding protein-19 (*RB19*) and eight housekeeping genes from Lauthier et al. [54]: glutathione peroxidase (*GPX*), 3-hydroxy-3-methylglutaril-CoA reductase (*HMCoAR*), pyruvate dehydrogenase component E1 subunit alfa (*PDH*), small *GTP*-binding protein Rab7 (*GTP*), Rho-like *GTP*-binding protein (*RHO1*), glucose-6-phosphate isomerase (*GPI*), superoxide dismutase B (*SODB*) and leucine aminopeptidase *(LAP*) were chosen for initial evaluation. Post screening, four of the ten targets (*TcMPX*, *SODB*, *GPX* and *GPI*) were discarded due to non-specific PCR amplification products and/or inconsistent sequence quality. The ratio of non-synonymous to synonymous amino acid changes (dN/dS) was calculated according to the Nei-Gojobori method [55] to infer relative selection pressures, where genes possessing a dN/dS ratio <1 meet the criteria for stabilizing selection for the conservation of metabolic function. Taylor & Fisher [56] recommend incorporating some loci with a dN/dS ratio of >1 in order to obtain sufficient sequence diversity. Chromosome location and other characteristics for each locus are shown in S2 Table. Additionally, we employed the FEL (Fixed Effects Likelihood) analyses [57], through the HiPhy application [58] via the Datamonkey web server [59] to infer selection pressure. Briefly, FEL analyses yields nonsynoymous (dN) and synonymous (dS) substitution rates on a per-site basis (assuming a significance level of 10% to all comparisons) for a given coding alignment and corresponding phylogeny through a maximum-likelihood approach.

#### MLST sequencing

PCR reactions were carried out in 20 uL reaction volumes containing 20 ng of DNA; 0.2 uM of each primer (S2 Table), 1U of Taq polymerase (Bioline, London, UK), 5X reaction buffer (Bioline, London, UK), 0.2 mM of each dNTPs (New England Biolabs, Hitchin, UK) and 1.5 mM MgCl2 solution (Bioline, London, UK). Amplification conditions for all targets were: 5 min at 94°C followed by 35 cycles of 94°C for 1 min; 55°C for 1 min, and 72°C for 1 min, with a final extension at 72°C for 5 mins. Products were visualized on 2% agarose gels, stained with ethidium bromide, followed by electrophoresis. Bi-directional sequencing was performed with Big Dye Terminator Cycle Sequencing V3.1 (Applied Biosystems, Foster City, CA) and ABI PRISM 3730 DNA Sequencing (Applied Biosystems, Foster City, CA) according to the manufacturer’s protocol. Sequences were aligned with BioEdit v7.1 [60] and DNASTAR Lasergene SeqMan v. 7.0 [61]. The presence of two coincident peaks at the same locus was verified in forward and reverse sequences and scored using IUPAC nomenclature.

#### MLST data analysis

Different approaches were used to analyze MLST data. Firstly, for each locus, typing efficiency (TE) and discriminatory power (DP) were assessed (MLSTest software, http://ipe.unsa.edu.ar/software) [62] to determine the resolution of individual markers. Following this, both diplotypic and haplotypic phylogenies were constructed. Diplotypic phylogenetic trees were initially constructed to investigate isolate diversity and enable concatenation across loci, as described by Yeo et al. [37] and Tavanti et al. [63], which renders diplotypic data suitable for use in distance based dendrograms. Briefly, individual loci between isolates can be considered either homozygous or heterozygous, assuming that *T*. *cruzi* is minimally diploid. For example, a homozygous locus scored as A (adenine) was modified by duplication to AA, and a heterozygous locus, for example Y (C or T, in accordance with IUPAC nomenclature), scored as CT, effectively creating a difference matrix across the panel. Phylogenies were also constructed from heterozygous SNPs were considered as average states. In more detail, the genetic distance between T and Y (heterozygosity composed of T and C) is considered as the mean distance between the T and the possible resolutions of Y (distance T-T = 0 and distance T-C = 1, average distance = 0.5 [18, 62].

Suitability of targets for inclusion into an MLST scheme was assessed via Incongruence Length Difference (ILD) tests, implemented through MLSTest 1.0 software using 1000 permutations [62]. This evaluates differences between expected and observed incongruences between loci in the context of random unstructured homoplasy [38]. Topological incongruence (TI), through MLSTest, was applied to the tree derived from concatenated loci to evaluated the number of fragment trees that are topologically incompatible. [64].

In a parallel approach, to investigate genetic exchange, gene haplotypes were inferred using PHASE v. 2.1 [65]. Genetic exchange within individual haplotypic gene phylogenies was examined by assessing allelic contributions to heterozygous isolates from putative homozygous donor genotypes with unambiguous phase. Genetic exchange within haplotypes was also examined in isolates with unambiguous phase, through RDP3 [66], in an attempt to detect allelic mosaics. RDP3 software implements an extensive array of methods for detecting and visualizing genetic exchange.

Lastly, MLST data were concatenated, testing all fragment combinations, using MLSTest to define the minimum combination of loci that resolves the maximum number of diploid sequence types (DSTs). Individual and concatenated phylogenies were generated initially with MrBayes, implemented through TOPALI v. 2.5 [67], using the best-fitting model, according to the Bayesian Information Criterion BIC. Two independent analyses were performed for 1.000.000 generations, sampling every 100 simulations (25% burn-in). Secondly, Neighbor Joining (NJ) method with uncorrected p-distances and ranch support was calculated by bootstrap set at 1,000 replications.

### Maxicircle (*COII*) data analysis

The TcI *COII* locus was amplified and sequenced according to Messenger et al. [41]. Nucleotide sequences per gene fragment are available at GenBank under accession numbers: MF781085-MF781124. Phylogenies were constructed implementing the substitution model based on the Akaike Information Criterion (AIC) in MEGA 6 [68]. To compare nuclear and mitochondrial topologies, Maximum-Likelihood (ML) phylogenies were constructed (T92+I model, Tamura 3-parameter) which assumes that a fraction of sites is evolutionarily invariable [68]. TcIII (CM17) and TcIV (Saimiri3 cl1, X10/610 cl5, ERA cl2 and 10R26) strains were included as outgroups (accession numbers: JQ581330.1, JQ581331.1, JQ581329.1, JQ581328.1 and JQ581327.1, respectively) [41].

### MLMT sequencing and data analysis

Twenty-five microsatellite loci were amplified as previously described by Llewellyn et al. [31] with some modifications (S3 Table). Markers were distributed across 11 chromosomes, including six groups of physically linked loci [69]. The following reaction conditions were implemented across all loci: a denaturation step of 4 mins at 95°C, 30 amplification cycles 95°C (20 s), 57°C (20 s), 72°C (20 s) with a final 20 mins elongation step at 72°C. Reactions were performed in a final volume of 10 μL containing, 1X ThermoPol Reaction Buffer (New England Biolabs (NEB, UK), 4 mM MgCl2, 34 μM dNTPs; 0.75 mM of each primer, 1 unit of Taq polymerase (NEB, UK) and 1 ng of genomic DNA. Five fluorescent dyes were used to label forward primers, 6-FAM & TET (Proligo, Germany), NED, PET & VIC (Applied Biosystems, UK). Allele sizes were determined using an automated capillary sequencer (ABI 3730, Applied Biosystems, UK), in conjunction with a fluorescently tagged size standard (GeneScan– 500 LIZ, Applied Biosystems, UK), and manually checked for errors in GeneMapper software v3.7 (Applied Biosystems, UK).

Microsatellite data were assessed in accordance with Lewellyn et al. [31]. Individual-level clustering defined by Neighbour-Joining (NJ) phylogenies (*D*_AS_: 1 –proportion of shared alleles at all loci/n) between microsatellite genotypes was calculated in MICROSAT v. 1.5 [70] under the infinite-alleles model (IAM). To accommodate multi-allelic genotypes (≥3 alleles per locus), a script was developed in Microsoft Visual Basic to generate random multiple diploid re-samplings of each Multilocus profile. A final pair-wise distance matrix was derived from the mean across multiple re-sampled datasets and used to construct a NJ phylogenetic tree in PHYLIP v3.67 [71]. Majority rule consensus analysis of 10,000 bootstrap trees was performed in PHYLIP v 3.6 by combining 100 bootstraps generated in MICROSAT v. 1.5 [70], each drawn from 100 randomly re-sampled datasets.

Population assignment with a prior assumption of subdivision by collection sites was estimated with the Bayesian clustering program Structure v. 2.3 [72]. We assumed the *admixture model* due to the lack of information regarding ancestry, with correlated allele frequencies (i.e. frequencies in different populations are similar as a consequence of migration or shared ancestry) [72]. Simulations were set at 10^6^ Markov Chain Monte Carlo (MCMC) interactions, with 2.5 x 10^5^ iterations as burn-in. Ten independent runs were performed for each value of K (that correspond to the number of groups, 2–10), as suggested by Pritchard et al. [72]. The most likely K value was estimated with the ΔK method [73].

An alternative approach to summarize genetic polymorphism was performed using a non-parametric approach, free from Hardy-Weinberg assumptions. Briefly, a *K*-means clustering algorithm, executed in ADEGENET [74] was used to identify the optimal number of ‘true’ populations, with reference to the BIC, which reaches a minimum when approaching the best support for assignment of isolates to the appropriate number of clusters. The relationship between clusters and the strains contained within them was evaluated using a discriminant analysis of principal components (DAPC), as described in Jombert et al. [74].

A single randomly sampled diploid dataset, generated using a custom Microsoft Visual Basic script to re-sample random multiple diploid combinations of each Multilocus profile, was used for all subsequent analyses, as described in Jombert et al [75]. Population genetic statistics were calculated considering strains assigned to their DAPC-derived population clusters. DTU-level genetic diversity was evaluated using sample size corrected allelic richness (Ar) in FSTAT v 2.9 [76]. Intra-population sub-clustering was calculated as mean pairwise *D*_AS_ values and associated standard deviations in MICROSAT v1.5 [70]. Sample size corrected private (population-specific) allele frequency per locus (PA/L) was calculated in HP-Rare [77]. Mean F_IS_, a measure of the distribution of heterozygosity within and between individuals, was calculated per population in FSTAT 2.9. *F*_IS_ varies between -1 (all loci are heterozygous for the same alleles) and +1 (all loci are homozygous for different alleles). DTU-level heterozygosity indices were calculated in ARLEQUIN v3.11 [78] and associated significance levels for p-values derived after performing a sequential Bonferroni correction to minimize the likelihood of Type 1 errors [79]. Population subdivision was estimated using pairwise F_ST,_ linearized with Slatkin’s correction, in ARLEQUIN v 3.11. Statistical significance was assessed via 10.000 random permutations of alleles between populations. Three different strategies were performed to group the samples and calculate pairwise F_ST_ values: i) using isolate collection locations to investigate local diversity, ii) to assess levels of gene flow between the five ecologically disparate biomes and, iii) investigate the role of host/vector specificity in the context of host movement and the distribution of TcI genotypes. Within-population subdivision was evaluated in ARLEQUIN v 3.11 [74] using a hierarchal analysis of molecular variance (AMOVA). A Mantel test for the effect of isolation by distance within populations (pairwise genetic vs. geographic distance) was implemented in GENAIEX 6.5 using 10,000 random permutations [80]. The association between host/vectors and genotypic clusters based on DAPC were calculated using contingency tables along with a Chi-squared test.

### Nucleotide sequence and read data accession numbers

Nucleotide sequences per gene fragment are available from GenBank under the accession numbers: MF615620-MF615679; MF615680-MF615739; MF615740-MF615799; MF615800-MF615859; MF615860-MF615919; MF615920-MF615979.

## Results

In our cohort of 78 isolates, 60 isolates were successfully characterized using all six MLST markers, 62 isolates by maxcicircle gene sequencing and 42 using MLMT markers. In particular, only twenty two isolates could be analyzed using all three methods. Furthermore, 50 more isolates were reassessed with MLMT, totaling 92 isolates considered for microsatellite analysis. S1 Table provides details of the typing methodologies applied to each particular isolate.

Six MLST markers were sequenced in 78 *T*. *cruzi* isolates, of which 60 consistently produced amplicons and sequences of acceptable quality. Concatenated gene fragments comprised a total of 2571 bp for each isolate. No single gene was able to differentiate all 60 isolates on the basis of TE and DP. Table 1 describes the level of diversity seen in each gene fragment; the number of polymorphic sites ranged from 10 (*RHO1*) to 1 (*LAP*). Typing efficiency (number of ST/number of polymorphisms) was variable among loci and the gene fragment distinguishing the highest number of genotypes per polymorphic site was *RB19* (TE = 2.25). In contrast, *CoAR* showed the lowest efficiency (TE = 1). *RHO1* demonstrated the highest DP (0.935) for our cohort; and *LAP*, the lowest (DP = 0.383).

**Table 1:** Properties of six *T*. *cruzi* MLST loci.

Targets	N^o^ of Polymorphic Sites	N^o^ of Genotypes	Typing Efficiency	Discriminatory Power	Ratio Of Nonsynonymous To Synonymous Changes
*CoAR*	9	9	1	0.529	0.16
*GTP*	7	10	1.42	0.646	0.032
*LAP*	1	3	3	0.383	0.30
*PDH*	5	10	2	0.549	0.073
*RB19*	4	9	2.25	0.829	0.014
*RHO1*	10	19	1.9	0.935	0.957

All fragments met the criterion for stabilizing selection (dN/dS<1) for conservation of metabolic function. FEL analyses detected signs of purifying (negative) selection in 10 sites across four gene fragments (*LAP* - 24th site; *RB19* - 35th and 63th sites; *RHO1* - 76th site; and *GTP* - 13th, 15th, 42th, 98th, 119th, 136th positions; p < 0.1).

A comparison of diplotypic phylogenies of individual gene trees revealed differences in topology and clustering between gene fragments (S1–S9 Figs). However, similarities did exist, most notably, the highly diverse loci *RB19* and *RHO1* possessed similar topologies (S8 Fig). Following concatenation of all six markers, DP increased to 0.997, differentiating 55 genotypes from 60 isolates.

The minimum number of loci needed to derive the maximum DP was assessed for all combination of fragments (2 loci to 6 loci) through MLSTest. A combination of 5 fragments: *CoAR-GTP-LAP-RB19-RHO1* (S10 Fig) also yielded a high DP (0.995), discriminating 53 genotypes (2 genotypes less than yielded by the use of all 6 MLST markers).

### MLST intraspecific diversity

Individual gene fragment trees revealed multiples polytomies in all six phylogenetic trees (S1–S9 Figs). Substantial congruences between the phylogenetic trees generated with SNP duplication (with Bayes) and Average State (with NJ) were observed (S1–S6 Figs), The two fragments with the most pronounced inconsistencies between Bayes and NJ were *PDH* and *RHO1* (S4 and S6 Figs).

S7–S9 Figs, show the comparison between the six gene trees. *RB19* and *RHO1* each produced a cluster corresponding to isolates from Atlantic Forest, Cerrado and Pantanal, which are mostly congruent. However, no two gene fragments showed completely identical topologies. The remaining loci (*CoAR*, *LAP*, *PDH* and *GTP*), which had lower TE and DP values, generally yielded trees that were less congruent. None of the gene fragments showed 100% congruence between their clusters.

Topological incongruence analyses revealed a mean of 2.86 incongruences per branch and 25% of branches with at least n-1 incongruent fragments. These correspond to moderate levels of incongruence (S11 Fig), where moderate incongruence was defined as being between 20 and 40% [64]. The ILD tests of discrepancies were no higher than expected, indicating that the combination of six gene fragments produces reliable branches (ILD = 0.05).

**Fig 2:**
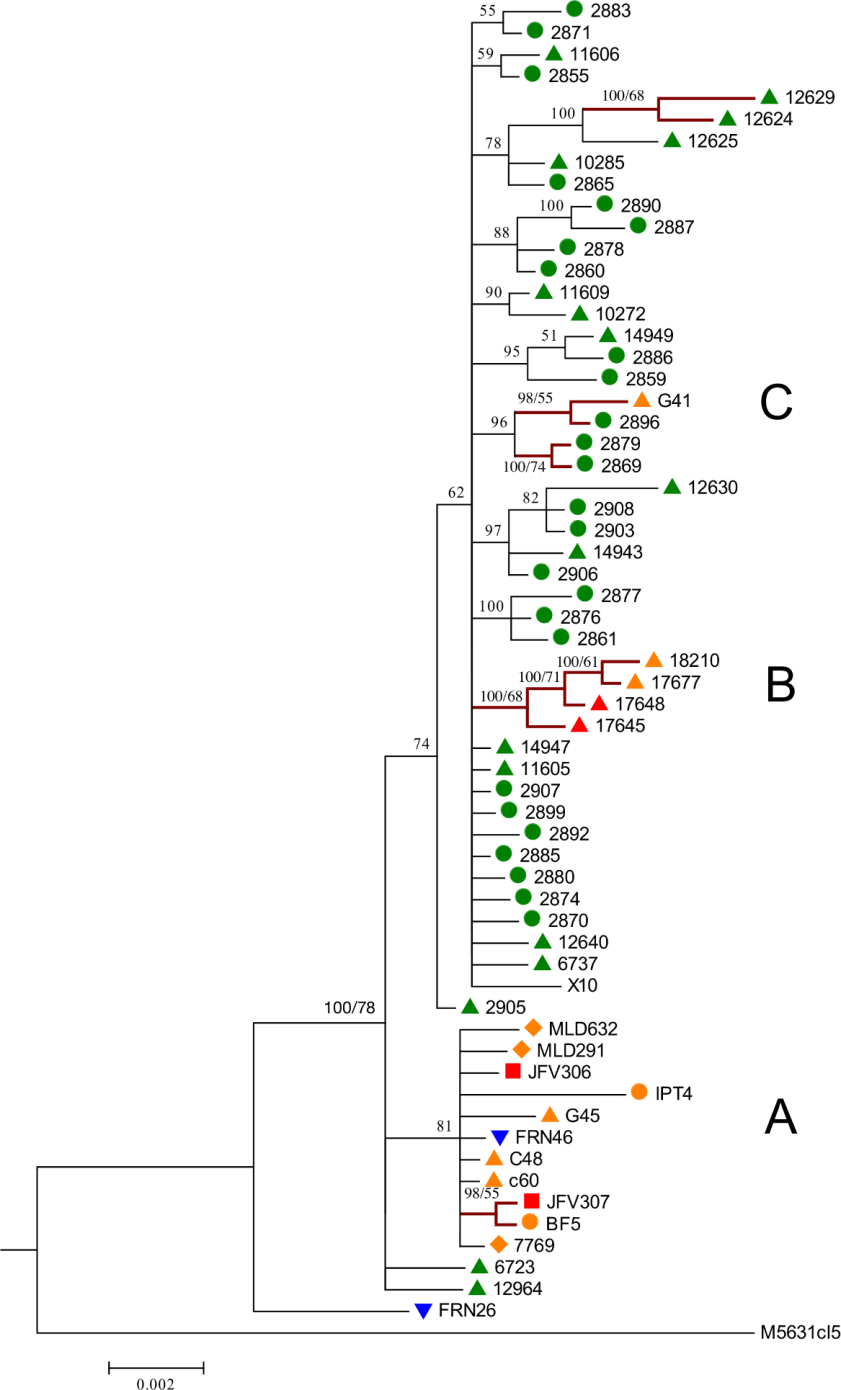
Bayesian inference based on the concatenation of 6 gene fragments from 63 TcI isolates. The highlighted clusters indicate congruence between SNPs duplication (Bayesian analysis) and Average State (NJ). Colors represent the biome of collection: green, Amazon; orange, Atlantic Forest; red, Cerrado; blue, Pantanal. Bullets correspond to mammal host or insect vector species: triangle, *Didelphis* spp.; square, *Chiroptera* spp.; circle, triatomine; diamond, primate; inverted triangle, rodent.

To evaluate intra-DTU diversity of TcI, phylogenies were inferred from the concatenated alignment of six gene fragments (Fig 2). Both, NJ and Bayesian methods produced similar results, although NJ analysis showed lower bootstrap values. Clusters with >50% support in both analyses are indicated. The presence of several sub-clusters was observed, revealing considerable intraspecific diversity within TcI and also similar genotypes circulating sympatrically over large geographical areas.

Specific phyloepidemiological observations are as follows. Cluster A grouped isolates originating from very distant localities including the Atlantic Forest, Cerrado and Pantanal biomes (BPP = 81% and bootstrap <50%). Of particular note, cluster A (Fig 2) lacked genotypes present in the Amazon, in congruence with results from maxicircle phylogenies (below). Also, similar genotypes were isolated from different species. For example, isolates from *Didelphis* spp, primates, chiroptera, one rodent, and triatomine bugs grouped within cluster A (Fig 2).

Amazonian isolates were genetically diverse and were mostly contained within a single clade (Fig 2). Cluster B (Fig 2) contained genotypes from the Atlantic Forest and Cerrado biomes, comprising an enormous geographical distance (~1.130 km). Interestingly, cluster C comprised isolates from distant biomes, Amazon and Atlantic Forest. Likewise, isolates from Abaetetuba (11609) and Cachoeira do Arari (10272), separated by vast geographical distances (~78.38 km), were grouped in the same cluster. Of note, a single isolate FRN26, *Oecomys mamorae* from the Pantanal, was genetically dissimilar from all other TcI strains and placed in different topological positions in the context of MLST and maxicircle phylogenies.

Although isolates were collected in different years and localities (S1 Table), no clear clustering by collection date or biome was apparent; however, statistical tests were not able to rule out the existence of some association. Details of these tests are described below.

### Haplotype analyses

**Fig 3:**
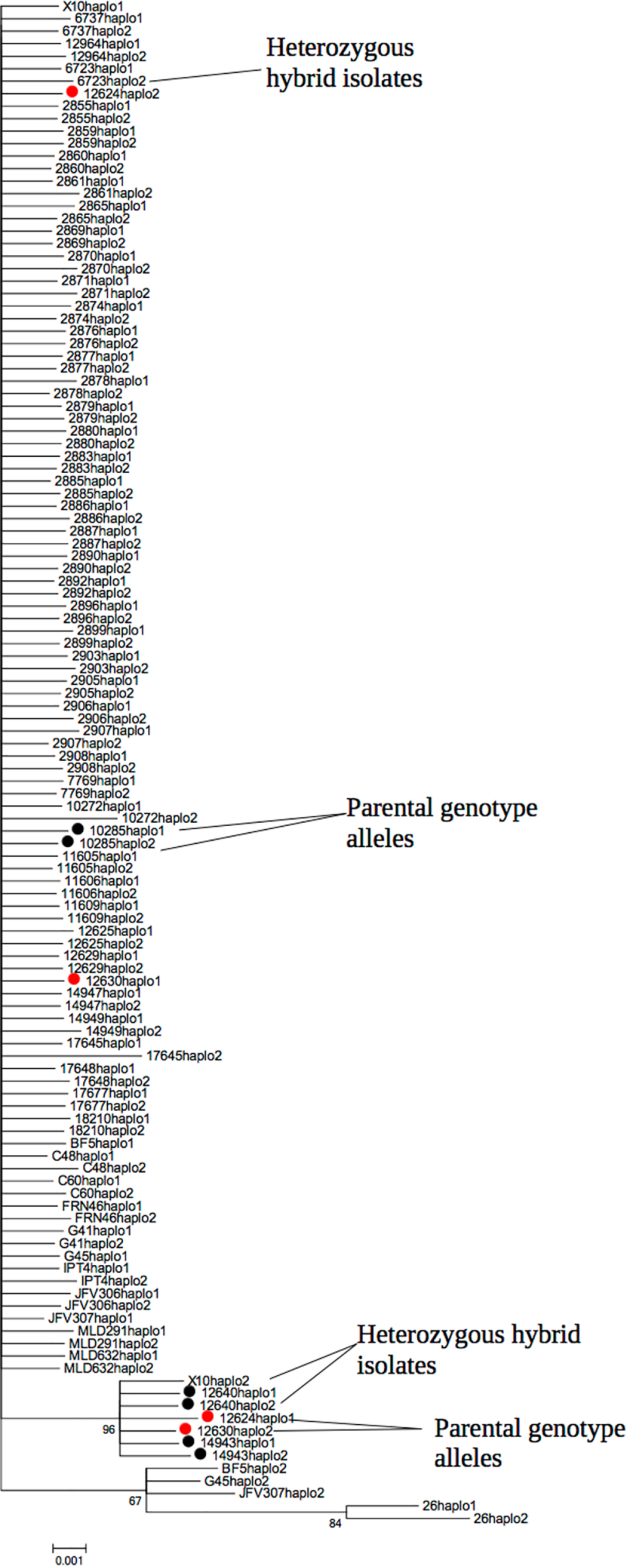
Haplotypic Bayesian Tree with *GTP* locus. Haplotypes inferred by PHASE V2.1. Red labels indicate heterozygous hybrid isolates (12630–12624) and black potential parental alleles (10285–14943).

Haplotype analysis, applied to nuclear loci, was used to generate phylogenies and investigate the allelic origins of heterozygous isolates from homozygous putative donor genotypes (Fig 3 and S12–S16 Figs). Here isolates with haplotypes present in two different genetically clusters that also contained respective homozygous donor isolate genotypes were considered potential hybrids.

Three genetic loci (*GTP*, *PDH* and *RB19*) revealed heterozygous isolates and allelic profiles that could be derived from homozygous genotypes (Fig 3, S12 and S13 Figs, respectively). In more detail, Fig 3 shows the *GTP* locus and alleles from homozygous donor isolates: 10285, haplotypes 1 and 2, in one clade; and 14943, haplotypes 1 and 2, in another. Within *GTP*, two isolates contain heterozygous allelic profiles, 12630 and 12624, corresponding to one allele from each homozygous donor. For *PDH*, five isolates contain heterozygous allelic profiles: 2892, 2896, 10285, 17645 and G41 (S12 Fig). The potential parental alleles for *PDH* were: 2879 and 2869, in one clade, and 2880, in another clade (S12 Fig). Similarly, for *RB19*, eight isolates showing potential genetic exchange were identified. The most plausible parental alleles for each putative hybrid are shown in S13 Fig, while the SNP profile for putative homozygous donors and the corresponding heterozygous profiles are shown in the S4–S6 Tables. Putative recombinants were different in *PDH*, *GTP*, and *RB19;* possibly indicating that there have been multiple genetic exchange events over time. Although we detect the signature of genetic exchange through heterozygous genotypes and their associated homozygous “donor” isolates, we observe no evidence of genetic exchange at the level of individual alleles, since allelic mosaics were not detected using RDP3 software.

### Mitochondrial analysis

**Fig 4:**
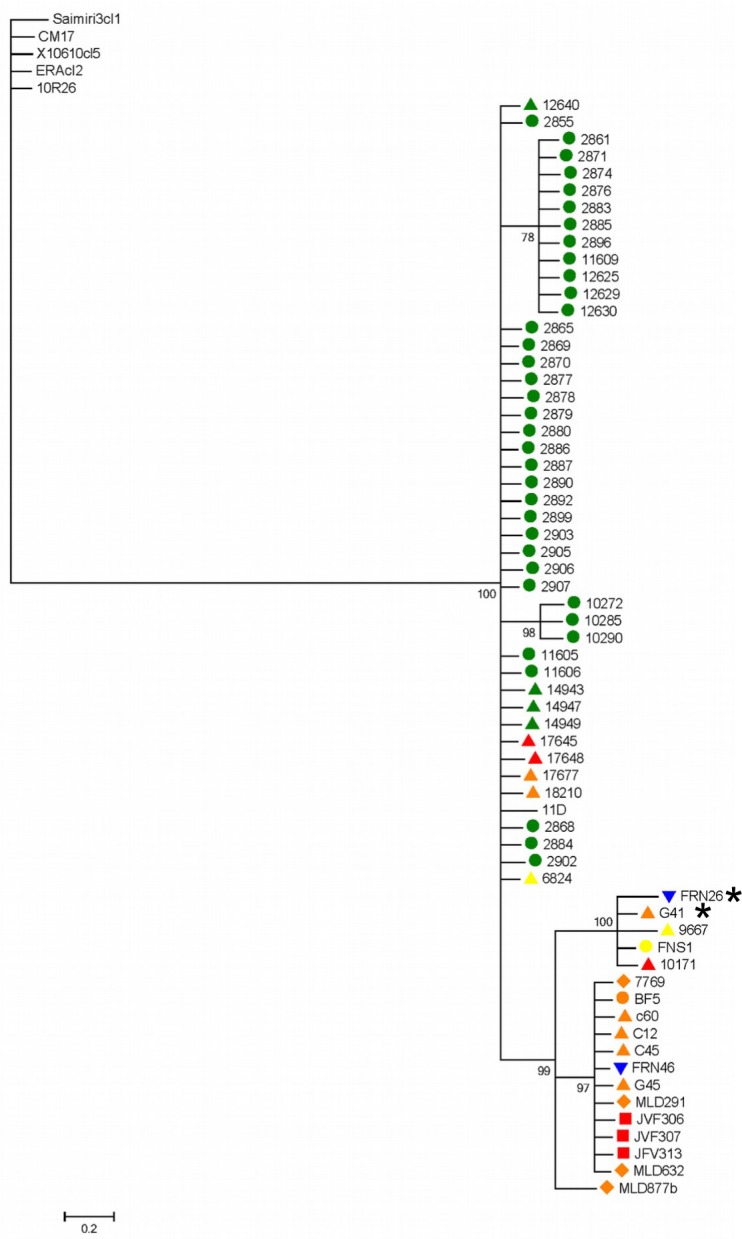
Maximum-Likelihood tree constructed from maxicircle sequences for 62 TcI Brazilian isolates. Colors represent the biomes where samples were obtained: Green, Amazon; orange, Atlantic Forest; red, Cerrado; blue, Pantanal; gray, reference sequences from Caatinga. Symbols correspond to strain host/vector: Triangle, *Didelphis* spp.; square, *Chiroptera* spp.; circle, triatomine; diamond, primate; inverted triangle, rodent.

Sixty two *COII* sequences produced a 449 bp alignment, 10 unique haplotypes and 64 polymorphic sites. Maximum-Likelihood trees (Fig 4) revealed two major clades and almost complete congruence with cluster A derived from concatenated MLST (S17 Fig). This cluster contains strains from the Atlantic Forest, Cerrado and Pantanal with the notable exclusion of Amazonian isolates (bootstrap = 100%). Interestingly, isolate FRN26 from the Pantanal, and isolate G41 from the Atlantic Forest formed a strongly supported sub-clade (bootstrap = 100%). In contrast, nuclear phylogenies grouped G41 with Amazonian isolates. Also of note, isolates within sub-clusters were highly homogeneous. Analyses with MLST and maxicircle were congruent in relation to the isolates of Amazon, in which they formed a separate group that included a cluster with isolates from Cerrado and Atlantic Forest (S17 Fig).

The presence of genetically identical mitochondrial sequences despite a mutation rate one order of magnitude greater than that of nuclear genes provides support for the occurrence of multiple mitochondrial introgression events (Fig 4 and S17 Fig). Additionally, these sequences correspond to geographically dispersed isolates, obtained from different biomes and hosts and vectors, further supporting the case for introgression.

### Microsatellite analysis

**Fig 5:**
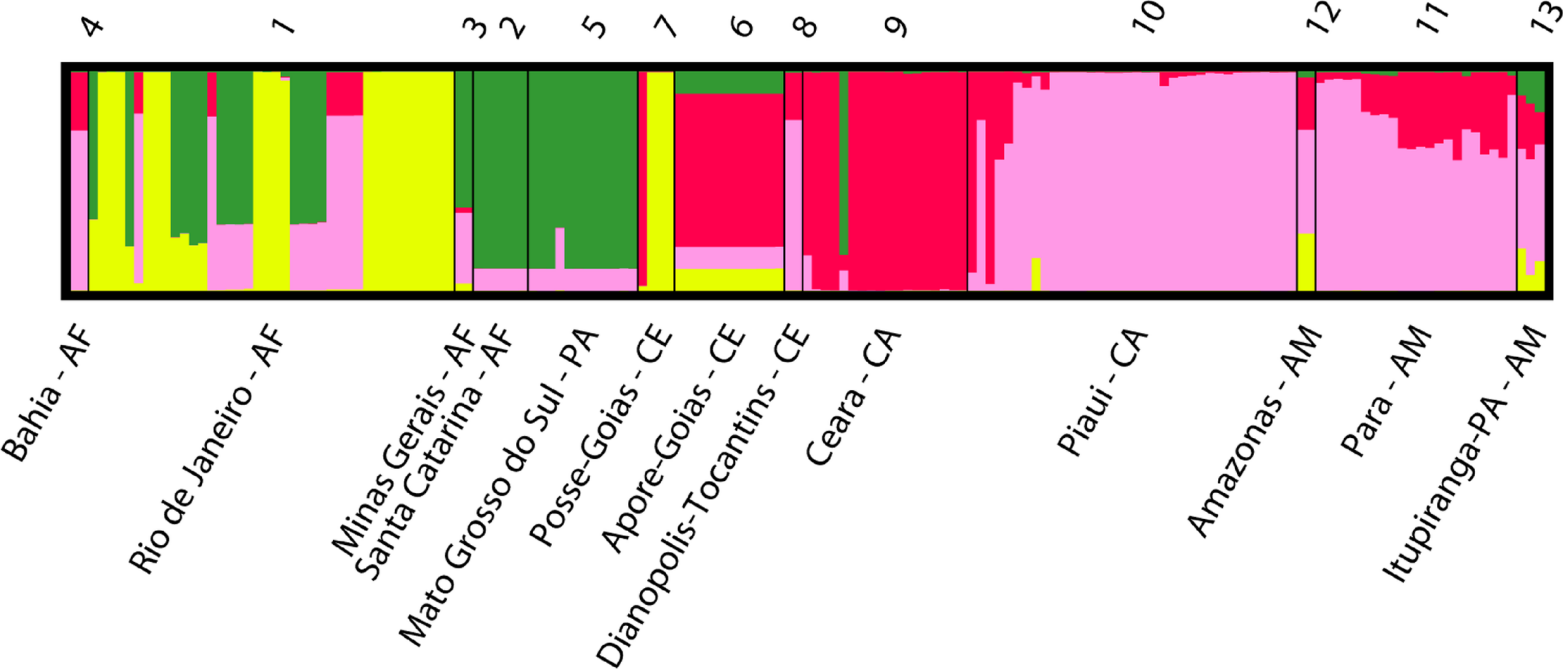
Population structure based on Bayesian clustering (K = 4) for 92 TcI isolates from five Brazilian biomes, genotyped for 25 microsatellite loci. Each number (above) represents the collection sites (below). Colours in vertical bars represent the proportion of the individual’s genome derived from four assumed clusters.

In total, 4595 alleles were identified, corresponding to 92 unique multilocus genotypes. Multiple (≥3) alleles were observed at 1.87% of markers. This is most likely attributable to aneuploidy in a small proportion of the loci (S7 Table). Bayesian clustering applied to 92 strains revealed the existence of four discrete phylogenetic groups without apparent association to the biome of origin (Fig 5). For example, isolates from Atlantic Forest clustered across three groups (Fig 5, yellow, green and pink label), in which specimens from the state of Rio de Janeiro are genetically similar to those from Posse, Goias (Cerrado biome) and specimens from the states of Minas Gerais and Santa Catarina clustered together with samples from Pantanal. Moreover, TcI specimens of the state of Bahia are genetically more similar to samples from the states of Piauí (Caatinga), Pará and Amazonas (Amazon) than to other samples from the Atlantic Forest biome. It is worth mentioning that, in general, samples from Cerrado, Caatinga and Amazon biomes were grouped together in two different groups (Fig 5, red and pink coloured groups).

**Fig 6:**
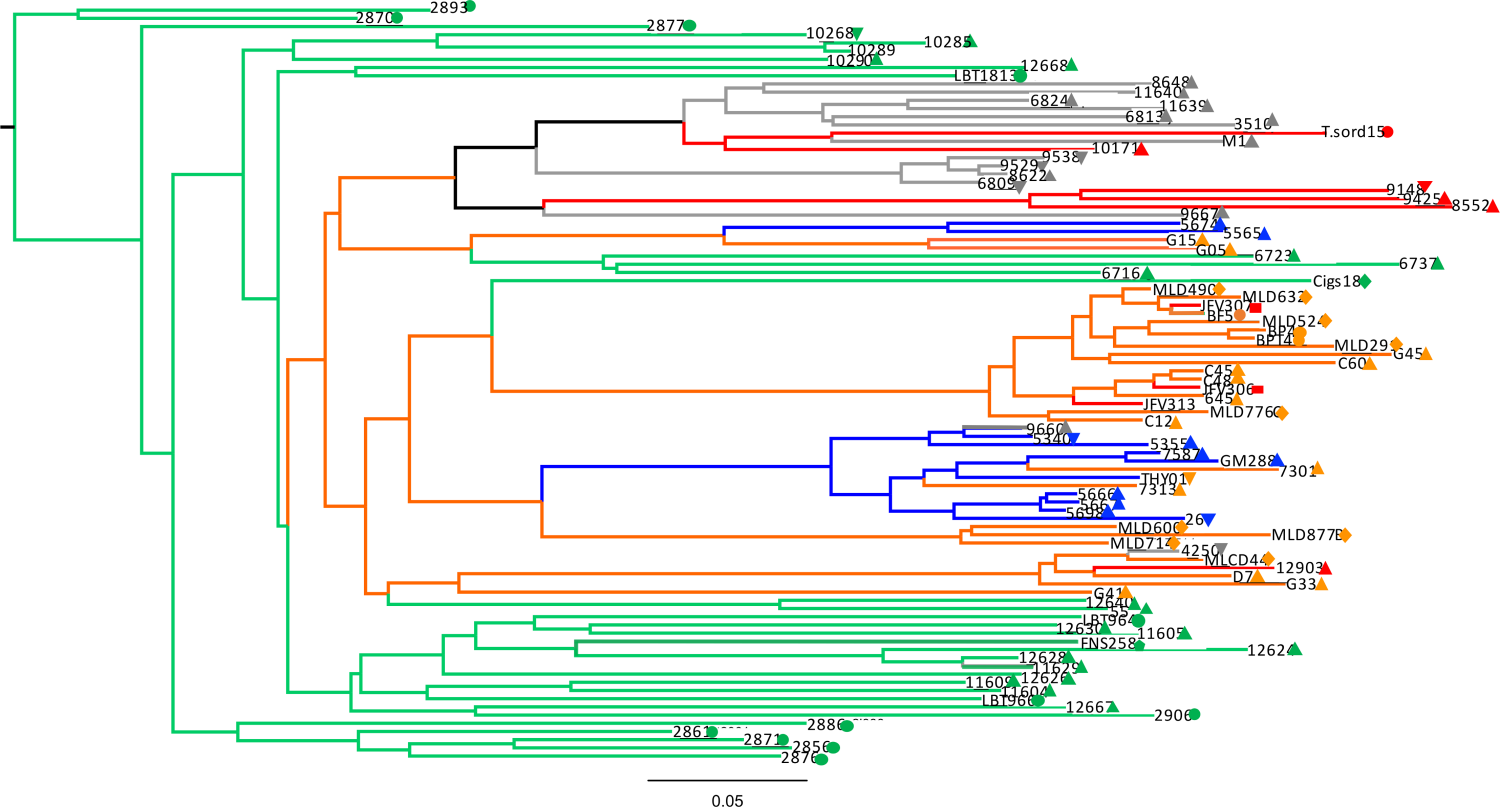
Unrooted neighbour-joining tree based on *D*_AS_ values between multilocus genotypes generated for 92 sylvatic Brazilian TcI strains. *D*_AS_ values were calculated as the mean across 1000 random diploid re-samplings of the data set. Branch colors represent the biomes where samples were collected: Green, Amazon; orange, Atlantic Forest; red, Cerrado; Bblue, Pantanal; gray, Caatinga. Symbols correspond to host/vector species: Triangle, *Didelphis* spp.; square, *Chiroptera* spp.; circle, Triatomine; diamond, Primate; inverted triangle, rodent; pentagon, dog; hexagon, Raccoon.

The DAPC analysis with the 92 strains yields five genetic clusters, evidenced by a slight ‘elbow’ in the distribution of the BIC values across optimal cluster numbers at K = 5, once 22 principal components (PCs) were retained and analyzed (representing 80% of the total variation) (S18 Fig). DAPC-derived populations were broadly congruent with patterns of nuclear clustering identified by NJ and Bayesian clustering analysis. The five DAPC clusters, showed in S1 Table, corresponded to: Population 1 that includes Caatinga (n = 13) and Cerrado (n = 4); population 2, Atlantic Forest (n = 4), Pantanal (n = 10) and Cerrado (n = 1); Population 3, Amazon (n = 30), Atlantic Forest (n = 6), Pantanal (n = 1) and Caatinga (n = 1); population 4, Atlantic Forest (n = 14) and Cerrado (n = 3) and population 5, the remaining parasites principally from opossums and primates in the Atlantic Forest (n = 14) and bats and an opossum in Cerrado (n = 3). Similarly, the NJ tree (Fig 6) reveals that parasites from the Atlantic Forest, Cerrado and Pantanal were generally admixed together. We observe no strict specific association between biomes, species or collection years and the clusters based on DAPC; however, the chi square contingency test (p<0.05) can not completely exclude an association between these clusters and host/vector species, collection biome or dates.

Cluster A, derived from MLST data, was congruent with one MLMT cluster, the equivalent maxicircle cluster (S17 and S19 Figs paired trees). The isolate G41 (Atlantic Forest), grouped with isolates from Amazonia for MLST but was grouped with Atlantic Forest isolates with MLMT analysis. Similarly, topological positions for FRN26/26 were different for MLST and maxcircle trees (S17 Fig paired trees)

Population genetic parameters were calculated for strains grouped *a priori* according to their biome of origin, as well as *a posteriori* DAPC cluster assignments (Table 2 and S8 Table). Consistent results are observed when strains are grouped according to DAPC-assigned clusters. Table 2 shows high levels of genetic heterogeneity in Amazon (DAPC population 3), as well as excess homozygosity, high numbers of private alleles per locus and a low standard deviation associated with D_AS_ value. *T*. *cruzi* strains from Atlantic Forest, Cerrado and Caatinga displayed similar, but lower levels of diversity, with comparatively lower numbers of private alleles per locus.

**Table 2:** Population genetic parameters for *a priori* sylvatic populations of TcI in Brazil.

Populations	G/N	PL	PA/L±SE	Ar±SE	D_AS_ ± SD	Ho	He	% PL He	% PL Hd	F_IS_±SE
Atlantic Forest	26/26	23	0.44 ± 0.12	3.61 ± 0.35	0.388 ± 0.188	0.37	0.46	26.1	43.5	0.212 ± 0.08
Amazon	32/32	25	0.97 ± 0.17	4.24 ± 0.38	0.457 ± 0.0846	0.42	0.51	0	36	0.194 ± 0.04
Pantanal	11/11	17	0.27 ± 0.14	2.46 ± 0.36	0.198 ± 0.157	0.35	0.31	23.1	5.9	-0.004± 0.16
Cerrado	09/09	19	0.36 ± 0.16	3.72 ± 0.74	0.482 ± 0.166	0.39	0.53	0	26.3	0.279 ± 0.12
Caatinga	14/14	20	0.13 ± 0.07	3.14 ± 0.46	0.341 ± 0.118	0.34	0.40	0	5	0.206 ± 0.09

N: number of isolates in population; G: number of multilocus genotypes (MLGs) per population based on microsatellite data of 25 loci analyzed; PL: number of polymorphic loci out of 25 loci analysed; PA/L: mean number of private alleles per locus ± SE, calculated in HP-Rare (Kalinowski, 2005); Ar: allelic richness as a mean over loci ± SE, calculated in FSTAT 2.9.3.2 (Goudet, 1995); D_AS_: mean pairwise allele sharing ± SD, calculated in MICROSAT v1.5d (Minch, 1997); Ho: mean observed heterozygosity across all loci, calculated in Arlequin v3.11 (Excoffier, 2005); He: mean expected heterozygosity across all loci, calculated in Arlequin v3.11 (Excoffier, 2005); %PL He: proportion of polymorphic loci showing a significant excess in heterozygosity after a sequential Bonferroni correction (Rice, 1989), calculated in Arlequin v3.11 (Excoffier, 2005); %PL Hd: proportion of polymorphic loci showing a significant deficit in heterozygosity after a sequential Bonferroni correction (Rice, 1989), calculated in Arlequin v3.11 (Excoffier, 2005); F_IS_: mean fixation index ± SE, calculated in FSTAT 2.9.3.2 (Goudet, 1995).

Three diverse populations (Atlantic Forest, Cerrado and Caatinga) were characterized by elevated standard deviations associated with D_AS_ values and positive F_IS_ values (Table 2). A hierarchical AMOVA demonstrated 83.1% of total genetic variation was present within populations, compared to 16.9%, among populations (p<0.0001 for both).

The observed subdivision between a priori populations suggests the existence of gene flow between *T*. *cruzi* from the Atlantic Forest and those of the Cerrado biome (F_ST_ = 0.067) (Table 3). Gene flow was also inferred to have occurred between *T*. *cruzi* populations of Caatinga and Cerrado (F_ST_ = 0.0982) (Table 3). The admixed character of these isolates was also supported by Bayesian assignment. More geographically-distant TcI populations display similar levels of subdivision, as observed between Caatinga and Pantanal, Caatinga and Atlantic Forest, Cerrado and Pantanal and Pantanal and the Atlantic Forest. *T*. *cruzi* isolates from the Amazon biome exhibited lower F_ST_ values than populations of all other biomes (Table 3). This observation is also supported by F_ST_ values calculated for the a posteriori populations (S9 Table).

**Table 3:** Pairwise F_ST_ values in a five-way comparison between *a priori* populations.

	Atlantic Forest	Amazon	Pantanal	Cerrado	Caatinga
Atlantic Forest	*				
Amazon	0.146**	*			
Pantanal	0.227**	0.172**	*		
Cerrado	0.067(p = 0.0156 ± 0.0037)	0.114**	0.267**	*	
Caatinga	0.216**	0.142**	0.347**	0.0982**	*

* 0.000

**P<0.001

At a local-level structure analysis (i.e. when samples were grouped using the collection site as prior information; Table 4), it is clear that some isolates from Atlantic Forest grouped with others from Cerrado and Pantanal due to the genetic similarity between samples of Rio de Janeiro and Possas, Goias (*F*_ST_ = 0.04), of Bahia and Tocantins (*F*_ST_ = 0.13), and of Santa Catarina and Mato Grosso do Sul (*F*_ST_ = 0.09). Similarly, samples from Cerrado (Piaui) and Amazon (Para) showed low levels of structure (*F*_ST_ = 0.09). The investigation of parasite population structure based on host taxonomy suggests that *Didelphis marsupialis* might play a role as the main disperser of TcI (S10 Table), since its overall pairwise *F*_ST_ values were lower than the others (*F*_ST_ ≤ 0.2; median = 0.15).

**Table 4:** Pairwise F_ST_ values for microsatellite data grouped according to the collection site.

	RJ	SC	MG	BA	MS	APGO	POGO	TO	CE	PI	PA	ITPA
RJ												
SC	0.26											
MG	0.29	0.56										
BA	0.24	0.57	0.56									
MS	0.24	0.09	0.47	0.48								
APGO	0.35	0.50	0.45	0.51	0.48							
POGO	0.04	0.39	0.46	0.33	0.35	0.43						
TO	0.28	0.58	0.52	0.13	0.50	0.49	0.41					
CE	0.22	0.39	0.42	0.31	0.34	0.34	0.22	0.39				
PI	0.18	0.28	0.32	0.27	0.23	0.35	0.23	0.31	0.20			
PA	0.16	0.25	0.27	0.25	0.22	0.30	0.20	0.26	0.16	0.09		
ITPA	0.17	0.37	0.31	0.33	0.33	0.39	0.23	0.37	0.23	0.19	0.13	
AM	0.22	0.45	0.5	0.41	0.44	0.45	0.31	0.37	0.23	0.19	0.13	0.30

False Discovery Rate (FDR) = 0,016. Yellow cells denote 0,016<p<0,05, blue cells denote 0,001<p<0,016 and, grey cells denote p<0,001

Finally, to determine the extent of spatial genetic structure, a Mantel test was conducted, demonstrating significant parasite isolation by distance across the sampled geographical range (*RXY =* 0.384; *P* = 0.01). (S20 Fig).

## Discussion

In this study we explored the genetic diversity of Brazilian TcI isolates, obtained from different vectors and mammal hosts, spanning 5 different ecological biomes. To this end, we analyzed data from six protein coding genes (MLST), 25 microsatellite loci and one mitochondrial locus. We observed substantial genetic diversity with no strict association of clusters with particular host/vector species or biomes. However, some degree of association to a cluster from MLMT was present in isolates from Amazon. No other noticeable relation between clusters and biomes was identified; nevertheless, statistical tests are consistent with the possibility of some form of association. In addition, we observed mitochondrial introgression events and evidence of intra-DTU genetic exchange. Previous works [29, 41, 81, 82] have studied the genetic diversity within DTU I using different methods encompassing nuclear and mitochondrial markers. However, this constitutes the first instance in which MLST analyses in combination with two other high resolution genetic markers (microsatellite and maxicircle sequencing) have been used to evaluate intra TcI diversity of isolates from Brazil.

The criteria for justifying the selection of MLST markers used were based broadly on Diosque et al. [18], and fragments were assessed, in order of importance, in terms of intra-DTU diversity (TE), genotype discrimination (DP) and statistical support in phylogenetic trees. MLST analysis using all six concatenated gene fragments discriminated 55 genotypes out of 60 isolates. The use of a combination of five fragments of concatenated genes (*CoAr*, *GTP*, *LAP*, *RHO1* and *RB19*) also proved to be a viable alternative to the six genes, discriminating 53 of a possible 60 isolates. However, in light of the slightly reduced discriminatory power, we recommend the use of all six gene fragments.

There was significant variation in TE and DP among tested loci (Table 1). The most variable loci were *RHO1* and *GTP* which possessed 17 and 13 polymorphic sites, respectively. This is in line with the observations made previously by Diosque et al. [18] and Ramirez et al. [36]. The *LAP* locus contained the least number of SNPs (4 polymorphic sites), in accordance with Ramirez et al. [36]. Some previous works have assessed *RB19*, *GPI*, *LAP* and *TR* and considered them non-informative when applied to typing schemes for cohorts spanning all six DTUs [18, 19, 36]. However, in the context of Brazilian isolates, *RB19* proved to be a highly informative marker for investigating intra-TcI diversity (TE = 1.9). Variation in TE is likely the result of selective pressures on individual loci, genetic drift or differences in mutation rates. Additionally, non-synonymous SNPs in MLST fragments, contributing to amino acid alterations, have previously been reported in *T*. *cruzi* [36, 37]. Here all gene fragments met the criteria for stabilizing selection (<1) for conservation of metabolic function. FEL analyses provided evidence that 10 sites in four of the six gene fragments are under purifying selection.

Bayesian and NJ analyses of the trees generated with each individual gene showed the presence of polytomies (S1–S6 Figs). One possible explanation for the existence of polytomies is the relatively small number of informative polymorphisms in the markers analyzed. This type of structure (Genetic Structure Type 2) was previously observed by Tomasini et al [64] when studying *A*. *fumigatus* through the application of MLST.

We generated phylogenetic trees with NJ to assess the robustness of our findings, since Bayesian analyses with SNP duplication can lead to artificially high bootstrap values [64]. Indeed, the support values were higher in the Bayesian analysis with SNP duplication; nevertheless, the clusters in the concatenated tree were mainly consistent between both analyses. Concatenation across loci by MLST has been successfully applied to many prokaryotic and eukaryotic organisms [22, 61, 63, 83, 84–86]. Nevertheless, when using this methodology, high levels of inbreeding or genetic exchange at particular loci may confound true phylogenetic relationships; therefore, in the presence of these effects, results must be interpreted cautiously. In this study, concatenation of six genes resulted in two main groups: the first included all isolates from Amazon and some representatives from Cerrado and Atlantic Forest (Fig 2, clusters B and C), and the second group included the remaining isolates from Atlantic Forest, Cerrado and part of Pantanal (Fig 2, cluster A). The epizootiological significance of these findings are discussed below.

### Nuclear genes, phylogenies and genetic exchange

The BPP values supporting those clusters that show incongruences varied widely between individual gene phylogenies. Similar patterns of incongruence have been previously observed in nuclear genes [37, 87]. Such incongruence, where isolates differ in topological positions, are a classical marker in populations that have undergone genetic exchange. To investigate further, haplotypic phylogenies were constructed for each genetic locus in order to define heterozygous isolates and their potential homozygous allelic donors. (Fig 3, S12–S16 Figs) The results indicate potential allelic recombinants in 3 of the 6 loci. Putative recombinant isolates possessed heterozygous allelic profiles, each present in two different homozygous putative donor isolates, situated in different phylogenetic clusters. Potential allelic recombinant isolates across 3 genes is suggestive of multiple genetic exchange events. PHASE is a Bayesian method for the reconstruction of haplotypes. It is generally considered one of the most accurate haplotype reconstruction methodologies. However, there are potential confounders, for example, population size and frequency of recombination have the potential to skew outcomes. Furthermore, one must be cautious when using PHASE to infer frequency of genetic exchange, as this is one of the assumptions of the method. Nevertheless, the presence of recombinants and potential “donor” genotypes inferred in three independent nuclear markers is confirmed by heterozygous and homozygous SNPs derived from nuclear sequences (S4–S6 Tables). Together, these observations constitute evidence for the presence of genetic exchange at the nuclear level. Population structure of *T*. *cruzi* is frequently regarded as clonal [88]. This model does not exclude genetic exchange, but considers it to be infrequent [89]. However, exchange across DTUs has been demonstrated using MLST [18, 36]; and intra-TcI genetic exchange in a single isolate has been observed in a cohort of Colombian samples [36]. Similarly, Messenger et al. [41] and Ramirez et al [82] observed multiple incongruence and introgression events within TcI on the basis of MLMT, MLST and maxicircle phylogenies, concluding that genetic exchange within DTU I is frequent. Genetic exchange is inferred in the current data set, however the frequency of genetic exchange is presently unknown and a topic of enthusiastic debate.

### Mitochondrial analysis

In comparison with nuclear genes, remarkably low levels of intra DTU *COII* diversity were observed. Paradoxically, the mutation rate of mitochondrial genes is generally considered one order of magnitude higher than that of nuclear genes [90]. The spectrum of reduced diversity observed in maxicircle clades is consistent with introgression events as also reported in different TcI populations in South America [36, 41]. Two major clades were observed, the first consisting of all samples from the Amazon biome, together with a few samples from other biomes. The second clade grouped all of the remaining samples. This pattern was congruent across both nuclear and mitochondrial loci, and is indicative of genetically discrete populations. MLMT analysis (below) suggests limited gene flow (F_ST_) between the Amazon biome and other studied areas. Although nuclear and mitochondrial phylogenies shared some topological characteristics, there were substantial incongruences between them. For example, isolate G41 clustered with Amazonian isolates at the nuclear level, but associated in mitochondrial phylogenies with isolates of non-Amazonian origin (S17 Fig). In the context of introgression, the discordance between nuclear and mitochondrial phylogenies is indicative of a prolonged and continuous association between populations from very distant localities [41]. This is consistent with the suggestion that genetic exchange in *T*. *cruzi* involves the independent exchange of kinetoplasts and nuclear genetic material [41]. Reciprocal nuclear genetic exchange among parasite strains undergoing mitochondrial introgression has not yet been detected, which may support an asymmetric, cryptic hybridization mechanism, or perhaps more likely, reflect the minor amount of nuclear genetic information sampled [81]. However, without the resolution of whole nuclear genome sequences, it is only possible to define the contributions of elements of meiosis, mitochondrial introgression and/or parasexual fusion [15, 82, 91]. The results presented here, include isolates from geographically distant sites (approximately 1790 km) and imply multiple introgression events occurring between different clades encompassing a large geographical area.

### Microsatellite analysis

MLMT, the most sensitive method for assessing diversity, identified 4 groups when collection sites were used to group TcI specimens (Fig 5) or 5 clusters when no prior clustering was imposed. Three groups draw attention, one with isolates originating from Caatinga (gray branch), another from Pantanal (blue branch) and a third, consisting of an admixture of Atlantic Forest and Cerrado (Fig 6, orange and red branch). The third group contained genotypes that occurred in primates, bats, *Didelphis* and *Rhodnius* spp., in agreement with mitochondrial phylogenetic topology. There was a tendency for TcI isolates to cluster with other locally obtained isolates, which may reflect a sampling bias or clonal expansion. However, when samples were grouped according to their collection sites (Table 4), the analysis revealed specific examples of similar genotypes present across nearby states. Examples include Santa Catarina (Atlantic Forest) and Mato Grosso do Sul (Pantanal), Bahia (Atlantic Forest) and Tocantins (Cerrado), and Piaui (Caatinga) and Pará (Amazon). In stark contrast, Amazon demonstrated significant intraspecific heterogeneity (Table 2 and Table 3) and clustering indices suggest that parasites from Amazon (DAPC population 3) have undergone long-term, undisturbed, sylvatic diversification. The relative lack of human impact, particularly in some municipalities in the state of Para, may account for allelic richness evolving over time in a biome with an abundance of host species. [39]. Interestingly, *D*_*AS*_ values from three diverse populations (Atlantic Forest, Cerrado and Caatinga) suggest the presence of intra-population sub-clusters, which is likely a consequence of the fragmentation due to intense human activity in these areas. Significant gene flow is observed over vast distances, for example between Cerrado and Atlantic Forest (Fig 1 and Table 3). The most parsimonious explanation is host movement, particularly aerial dispersion with bats, as exemplified over large distances in African clades of *Trypanosoma* sp. [92]. In South America, bats are known to harbor diverse trypanosome genotypes [92, 93], but their role in biogeography and dispersion is not fully understood. Unfortunately, TcI samples from Chiroptera species were collected from a single location (in Cerrado). A much more comprehensive effort to study Tc1 isolates in Chiroptera would be of interest to adequately address the nature of their role in dispersal in Brazil. Notwithstanding, we observed that *D*. *marsupialis* acts as a disperser of TcI genotypes across different biomes [94], this is evidenced by generally low *F*_*ST*_ values in pairwise comparisons with samples obtained from other hosts (*F*_ST_ ≤ 0.2, S10 Table). Isolates from Atlantic Forest, Amazon and Cerrado showed significantly low heterozygosity levels, which may be due to gene conversion or under sampling used in the study. In this case, processes such as inbreeding are expected to shape the genetic background of populations [94]. Indeed, isolates from the Amazon biome presented low gene flow and moderate levels of inbreeding (*F*_IS_ = 0.194 ± 0.04), relatively to other biomes, indicating a degree of genetic isolation. (Table 2).

### Epizootiology of Brazilian TcI

Our analyses of three classes of genetic markers revealed broadly similar patterns of intra-DTU diversity in Brazil. MLST and maxicircle marker analysis yielded two principal phylogenetic groups. One included all isolates from the Amazon region, with representatives from Cerrado and Atlantic Forest (Fig 2, clusters B and C). The second group included all other isolates from Atlantic Forest, Cerrado and part of Pantanal (Fig 2, cluster A). MLMT analysis comprising fast evolving markers, as expected, revealed the most diversity, five discrete populations and variable amounts of gene flow and fragmentation indicators. Among all biomes it is evident that Amazon harbors the most extensive diversity and comparatively low gene flow. High diversity and low fragmentation indicate a biome exposed to less ecological pressure and undisturbed sylvatic diversification. Generally, there was no clear evidence of specific host/vector associations. In particular, similar genotypes were represented in different vector/host species. For example, genotypes represented in cluster A (Fig 2) consisted of closely related genotypes observed in a diversity of hosts species including didelphids, rodents, chiroptera, primates and triatomines scattered across diverse municipalities within the Atlantic Forest, Cerrado and Pantanal biomes. Additionally, this cluster included hosts whose habitat is principally arboreal, with *Didelphis* spp occupying all strata. The presence of *Didelphis* spp. in all clades and low associated *F*_ST_ values (S10 Table) is compatible with the hypothesis that they are bioaccumulators of multiple genotypes [83, 94], they are highly permissive to infection and are known to move between all ecological strata from terrestrial to arboreal. The genealogical relationship of isolates in cluster A in MLST was preserved across MLMT and mitochondrial analyses (S17 and S19 Figs).

Evidence from all markers reveals that similar genotypes are found across vast geographical distances, over ecological barriers, diverse habitats, and different hosts species. Noticeably, isolates G41 (Atlantic Forest) and 2896, from Belem in the Amazon biome (Figs 1 and 2), have identical genotypes. Other examples include isolates 10272 and 11609, which possess identical genotypes despite being separated by the *Marajo bay* (a distance of 4500 km); and isolates from Belem (2855) and Abaetetuba (11606), which are genetically homogenous despite vast geographic separation. Human activity is likely to have an impact on the dispersal of genotypes. A case in point is provided by Combu and Murucutu, which are two island localities situated in the municipality of Belem (Amazon) that are sparsely occupied by humans and used primarily for açaí production [53]. They form a robust enzootic transmission cycle, and remote human infections are acquired by unwitting transport of infected triatomines in açai baskets [53]. Comparatively high indicators of gene flow between other biomes inferred by MLMT analysis are also compatible with the influence of human activity that may facilitate gene flow. Lima and collaborators [42] using MLMT, applied to Brazilian TcI, observed that isolates from Atlantic Forest and the Amazon formed distinct and separate clusters. Their proposition was that geographic distance separating biomes was the likely explanation for topological features. However, in this work, through the application of MLST, MLMT and maxicircle analysis, we find not only localized diversity but also genetic homogeneity over large distances. In summary, this study included a large number of samples and revealed extensive intra DTU diversity, an absence of strict associations to host/vector species, and similar genotypes circulating over vast areas. We provide evidence of genetic exchange based on phylogenetic incongruence among loci, haplotypic analysis of nuclear markers and also mitochondrial introgression. It is likely that gene flow between biomes is influenced by the movement of mammals and also facilitated by human activity.

## Supporting information

S1 Fig Neighbor Joining tree (A) and Bayesian tree (B) based in *COAR* gene fragment.
(PDF)

S2 Fig Neighbor Joining tree (A) and Bayesian tree (B) based in *GTP* gene fragment.
(PDF)

S3 Fig Neighbor Joining tree (A) and Bayesian tree (B) based in *LAP* gene fragment.
(PDF)

S4 Fig Neighbor Joining tree (A) and Bayesian tree (B) based in *PDH* gene fragment.
(PDF)

S5 Fig Neighbor Joining tree (A) and Bayesian tree (B) based in *RB19* gene fragment.
(PDF)

S6 Fig Neighbor Joining tree (A) and Bayesian tree (B) based in *RHO1* gene fragment.
(PDF)

S7 Fig Trees generated with individual fragments using Bayesian analysis. (A) *CoAR*, (B) *GTP*.
(PDF)

S8 Fig Trees generated with individual fragments using Bayesian analysis. (A) *RHO1*, (B) *RB19*.
(PDF)

S9 Fig Trees generated with individual fragments using Bayesian analysis. (A) *PDH* and (B) *LAP*.
(PDF)

S10 Fig MLST: Reduced 5 loci combination scheme. (PDF)

S11 Fig Topological incongruence based on the six concatenate gene markers. (PDF)

S12 Fig Haplotypic Bayesian Tree with *PDH* locus. (PDF)

S13 Fig Haplotypic Bayesian Tree based on *RB19* locus. (PDF)

S14 Fig Haplotypic Bayesian Tree based on *CoAR* locus. (PDF)

S15 Fig Haplotypic Bayesian Tree based on *LAP* locus. (PDF)

S16 Fig Haplotypic Bayesian Tree based on *RHO1* locus. (PDF)

S17 Fig Comparison between (A) MLST and maxicircle trees (B).
(PDF)

S18 Fig Nuclear genetic clustering among 92 Brazilian sylvatic TcI strains. (PDF)

S19 Fig Comparation between (A) MLST and (B) MLMT trees.
(PDF)

S20 Fig Nuclear spatial genetic analysis of *T*. *cruzi* I isolates from five Brazilian biomes. (PDF)

S1 Table *Trypanosoma cruzi* I isolates used in the study. (PDF)

S2 Table MLST gene targets. (PDF)

S3 Table Panel of microsatellite loci and primers. (PDF)

S4 Table SNP data of isolates for *RB19*. (PDF)

S5 Table SNP data of isolates for *PDH*. (PDF)

S6 Table SNP data of isolates for *GTP*. (PDF)

S7 Table Complete dataset of 25 microsatellite markers. (PDF)

S8 Table Population genetic parameters for a posteriori sylvatic population of TcI in Brazil. (PDF)

S9 Table F_ST_ values in a five- way comparison between a posteriori population. (PDF)

S10 Table Pairwise F_ST_ values for microsatellite data grouped according to the parasites’ hosts. (PDF)
