# Supplementary material for: Dissecting the phyloepidemiology of Trypanosoma cruzi I (TcI) in Brazil by the use of high resolution genetic markers
Source: PLoS Negl Trop Dis. 2018 May 21;12(5):e0006466. doi: 10.1371/journal.pntd.0006466 (PMC5983858; doi:10.1371/journal.pntd.0006466)
Supplement: S2 Fig — Neighbor Joining tree (A) and Bayesian tree (B) based in GTP gene fragment. (PDF) [file pntd.0006466.s002.pdf]

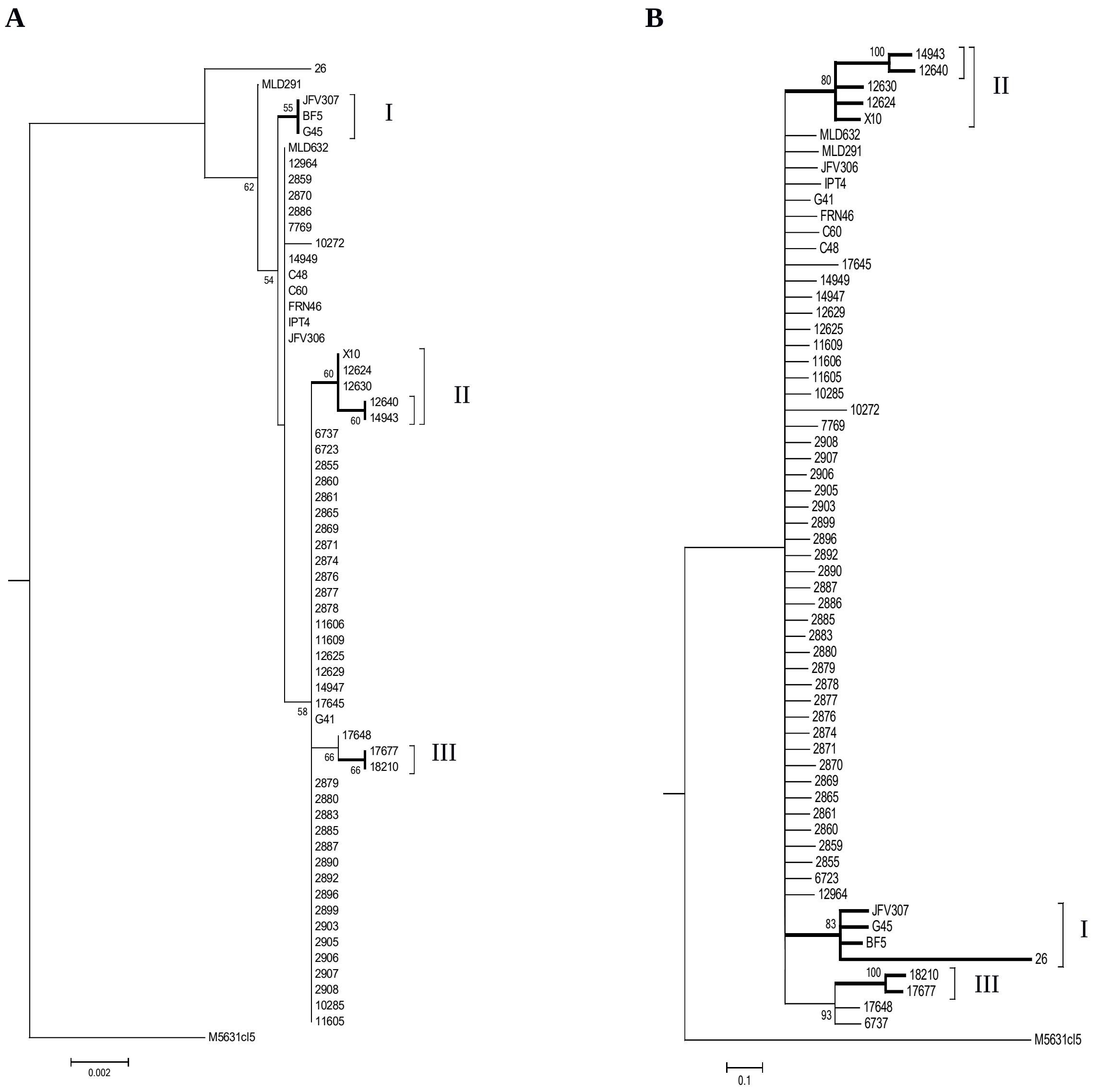

S2 Fig. Neighbor Joining tree (A) and Bayesian tree (B) based in *GTP* gene fragment. Roman numerals identify clusters that were consistent across both analyses.
