# Supplementary material for: Dissecting the phyloepidemiology of Trypanosoma cruzi I (TcI) in Brazil by the use of high resolution genetic markers
Source: PLoS Negl Trop Dis. 2018 May 21;12(5):e0006466. doi: 10.1371/journal.pntd.0006466 (PMC5983858; doi:10.1371/journal.pntd.0006466)
Supplement: S6 Fig — Neighbor Joining tree (A) and Bayesian tree (B) based in RHO1 gene fragment. (PDF) [file pntd.0006466.s006.pdf]

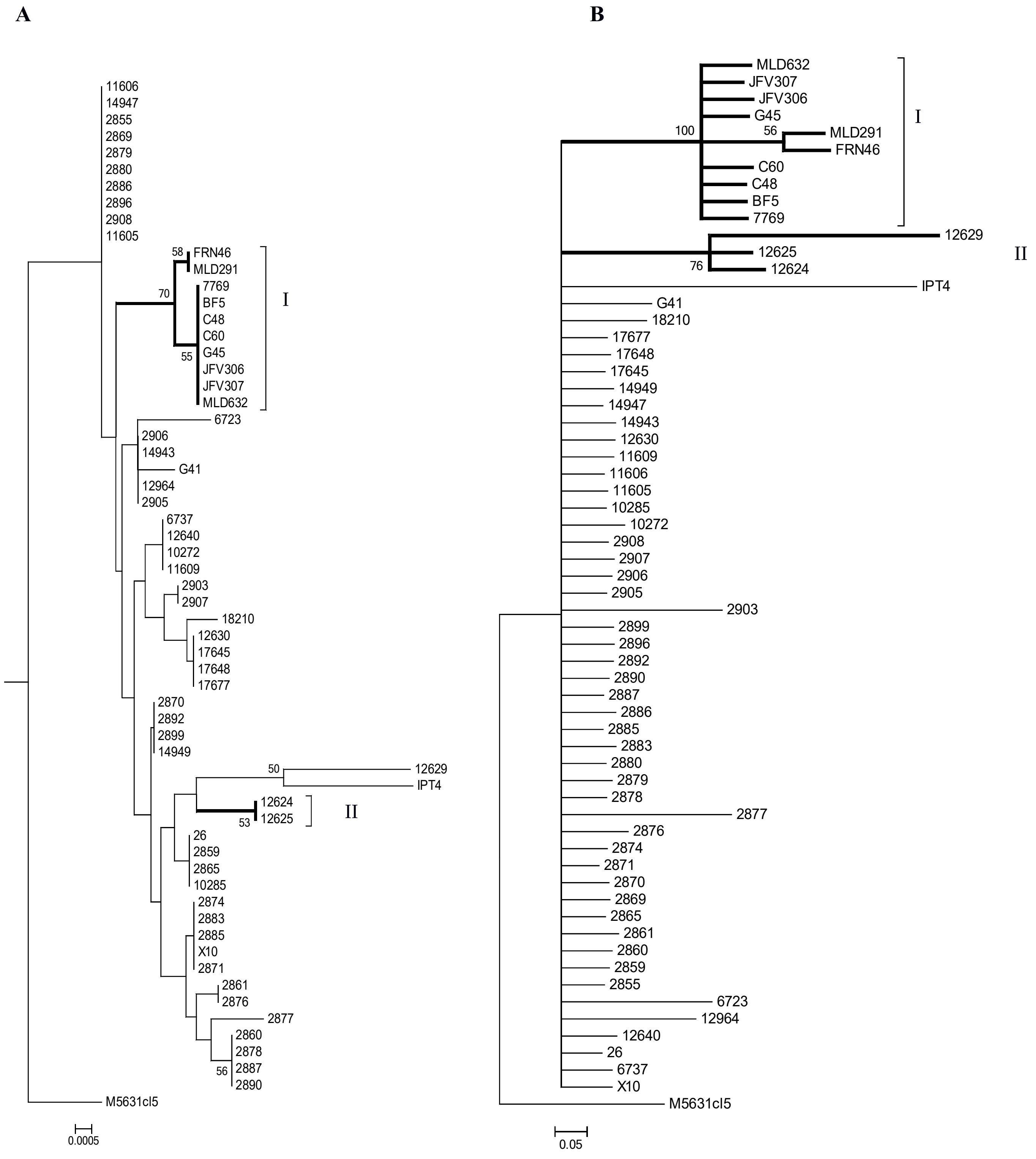

S6 Fig. Neighbor Joining tree (A) and Bayesian tree (B) based in *RHO1* gene fragment. Roman numerals identify clusters that were consistent across both analyses.
