# Supplementary material for: Dissecting the phyloepidemiology of Trypanosoma cruzi I (TcI) in Brazil by the use of high resolution genetic markers
Source: PLoS Negl Trop Dis. 2018 May 21;12(5):e0006466. doi: 10.1371/journal.pntd.0006466 (PMC5983858; doi:10.1371/journal.pntd.0006466)
Supplement: S7 Fig — Trees generated with individual fragments using Bayesian analysis. (A) CoAR, (B) GTP. (PDF) [file pntd.0006466.s007.pdf]

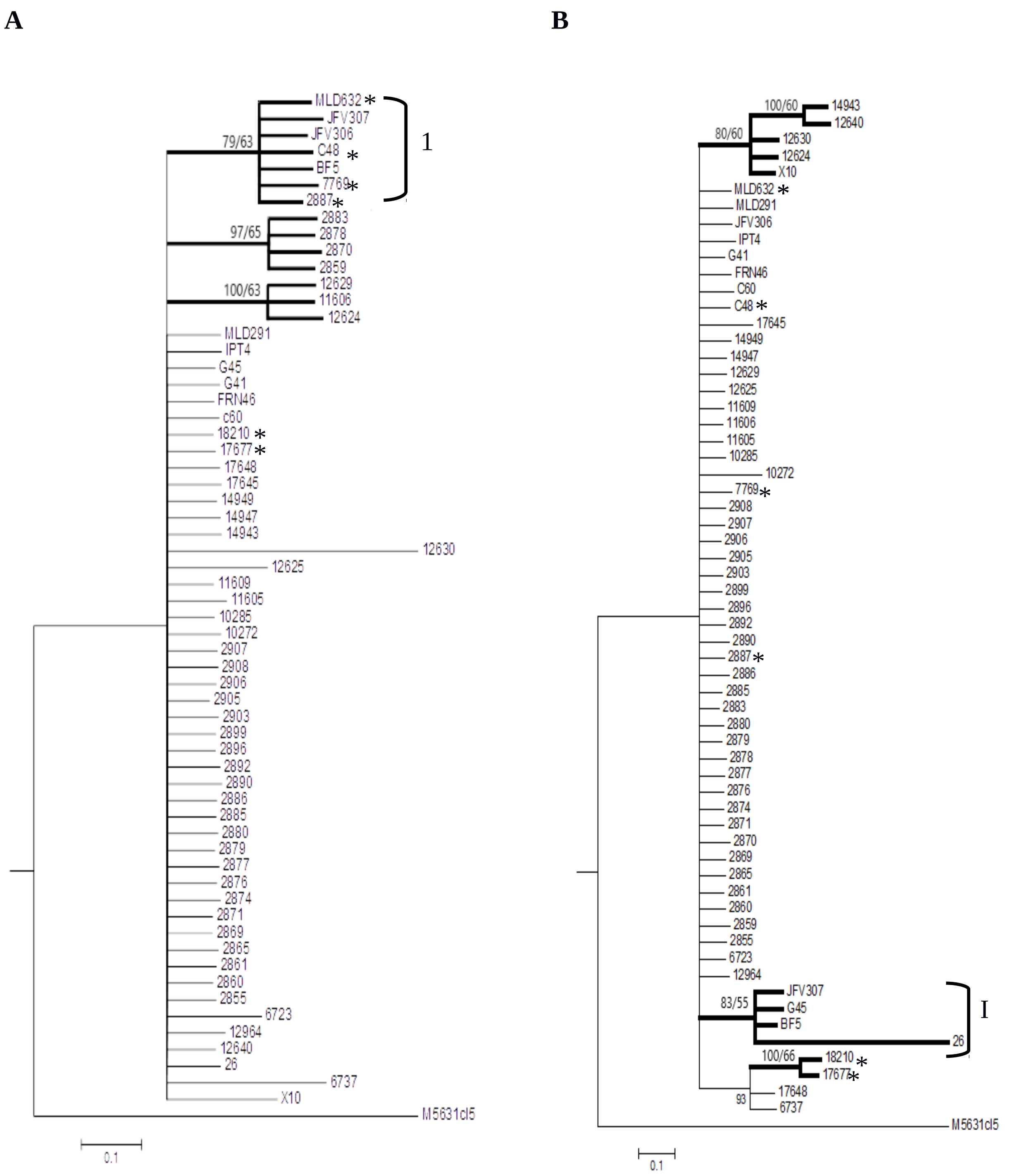

S7 Fig. Trees generated with individual fragments using Bayesian analysis. (A) *CoAR*, (B) *GTP*. Highlighted clusters in each tree indicate agreement between Bayesian analyses and NJ, for which both bootstrap values are shown. Roman numerals identify partial congruence across both markers and asterisks signal outstanding incongruences.
