# Supplementary material for: Dissecting the phyloepidemiology of Trypanosoma cruzi I (TcI) in Brazil by the use of high resolution genetic markers
Source: PLoS Negl Trop Dis. 2018 May 21;12(5):e0006466. doi: 10.1371/journal.pntd.0006466 (PMC5983858; doi:10.1371/journal.pntd.0006466)
Supplement: S9 Fig — Trees generated with individual fragments using Bayesian analysis. (A) PDH and (B) LAP. (PDF) [file pntd.0006466.s009.pdf]

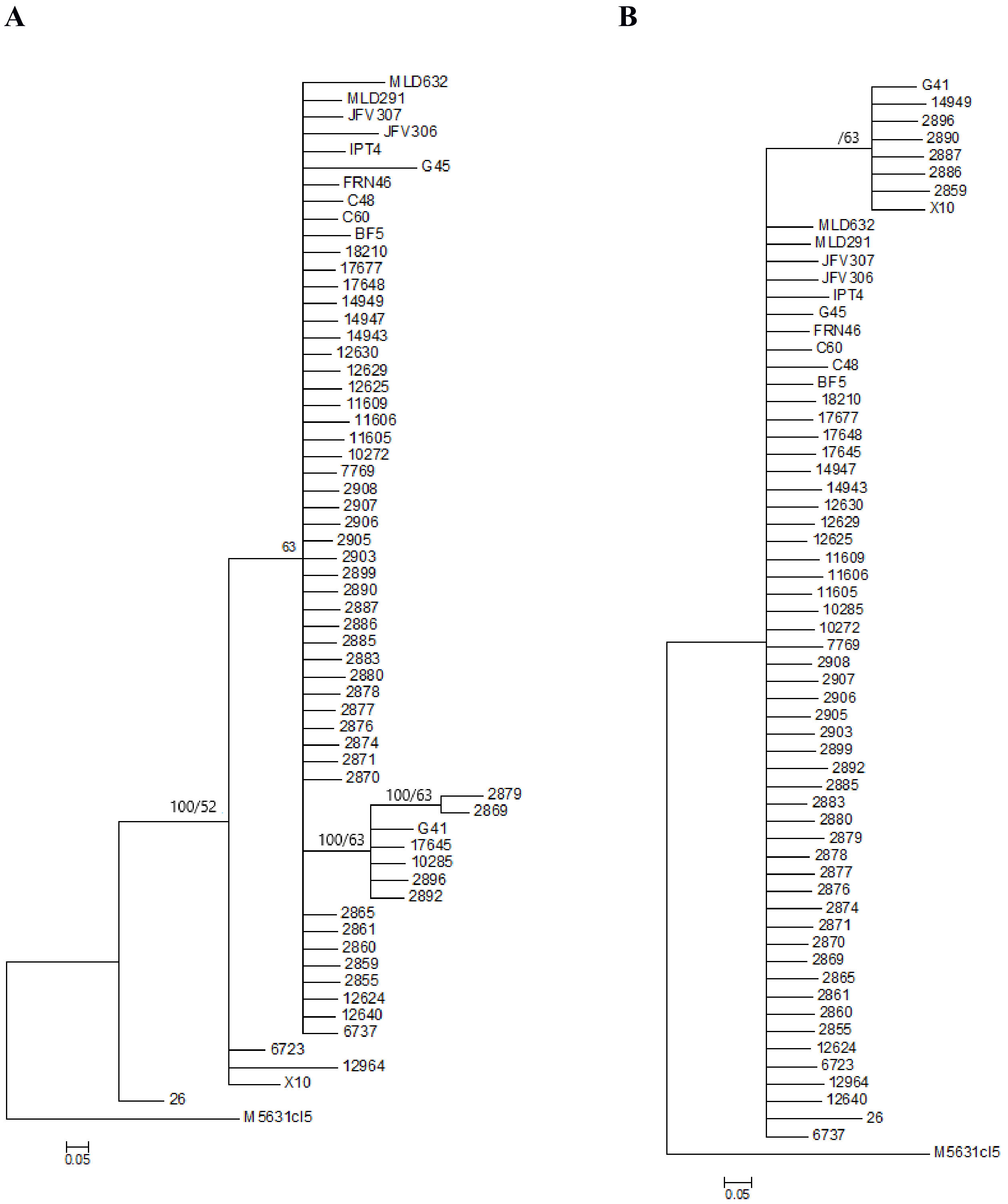

S9 Fig. Trees generated with individual fragments using Bayesian analysis. (A) *PDH* and (B) *LAP*. Bootstrap values (>50%) for Bayesian analysis and NJ are shown. Substantial incongruence were observed.
