# Supplementary figures and images for: Dissecting the phyloepidemiology of Trypanosoma cruzi I (TcI) in Brazil by the use of high resolution genetic markers
Source: PLoS Negl Trop Dis. 2018 May 21;12(5):e0006466. doi: 10.1371/journal.pntd.0006466 (PMC5983858; doi:10.1371/journal.pntd.0006466)

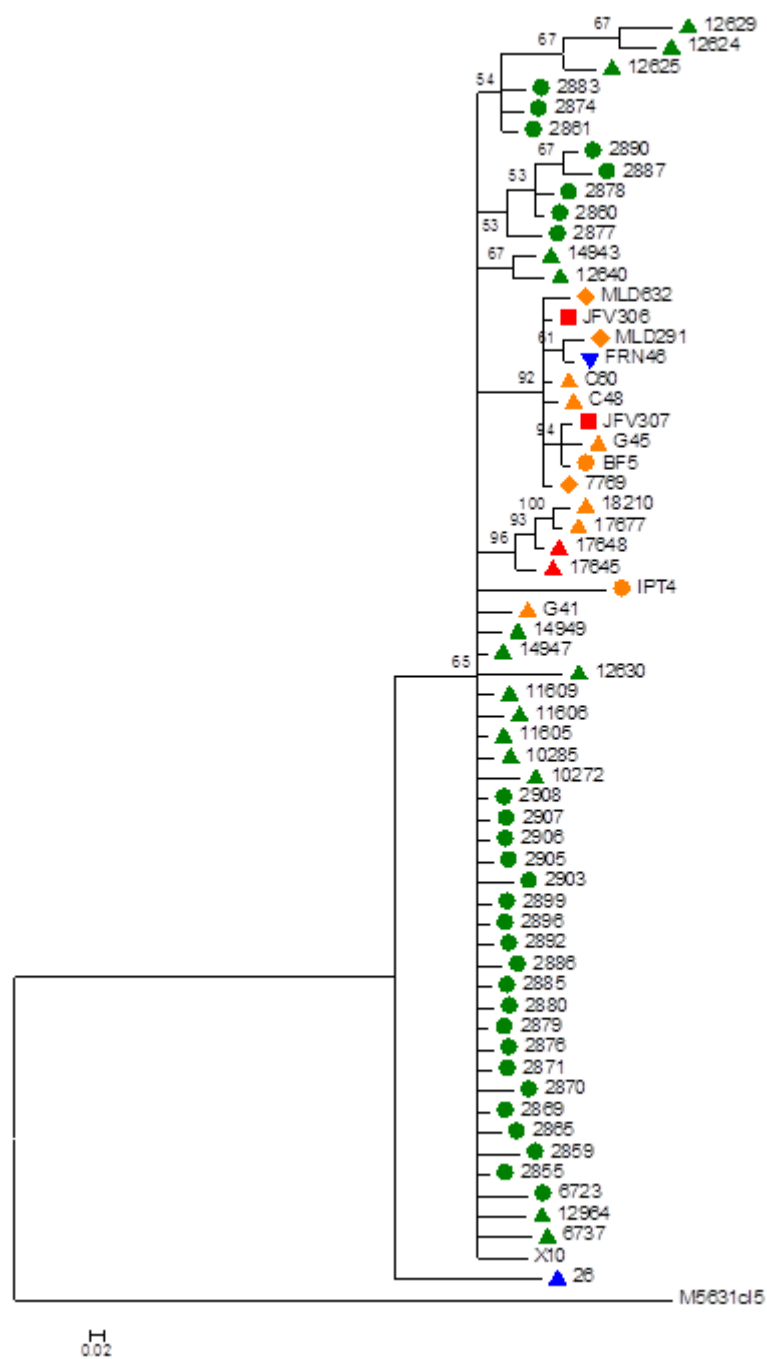

**S10 Fig. MLST: Reduced 5 loci combination scheme (*CoAR-GTP-LAP-RHO1-Rb19*)**

Supplement: S10 Fig — (PDF) [file pntd.0006466.s010.pdf]

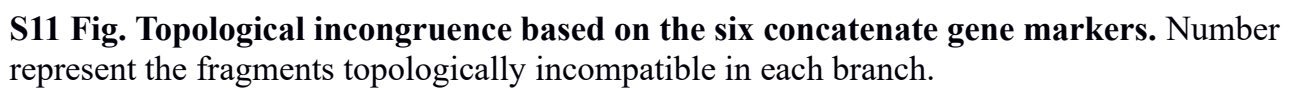

Supplement: S11 Fig — (PDF) [file pntd.0006466.s011.pdf]

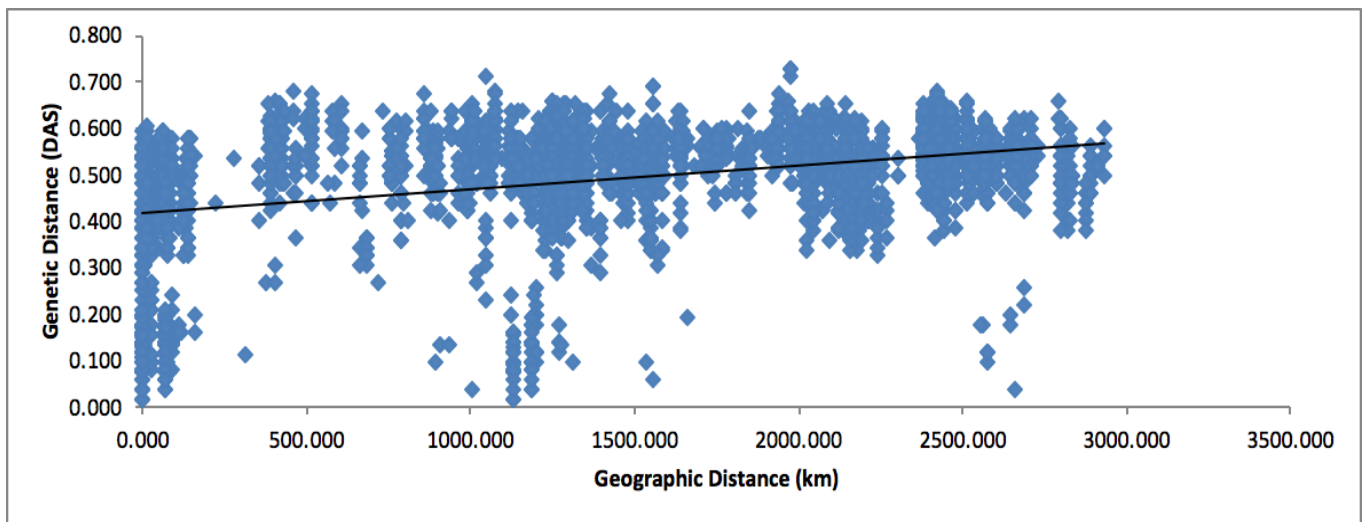

**S20 Fig. Nuclear spatial genetic analysis of *T. cruzi* I isolates from five Brazilian biomes.**

Supplement: S20 Fig — (PDF) [file pntd.0006466.s020.pdf]
