# Supplementary material for: Dissecting the phyloepidemiology of Trypanosoma cruzi I (TcI) in Brazil by the use of high resolution genetic markers
Source: PLoS Negl Trop Dis. 2018 May 21;12(5):e0006466. doi: 10.1371/journal.pntd.0006466 (PMC5983858; doi:10.1371/journal.pntd.0006466)
Supplement: S13 Fig — (PDF) [file pntd.0006466.s013.pdf]

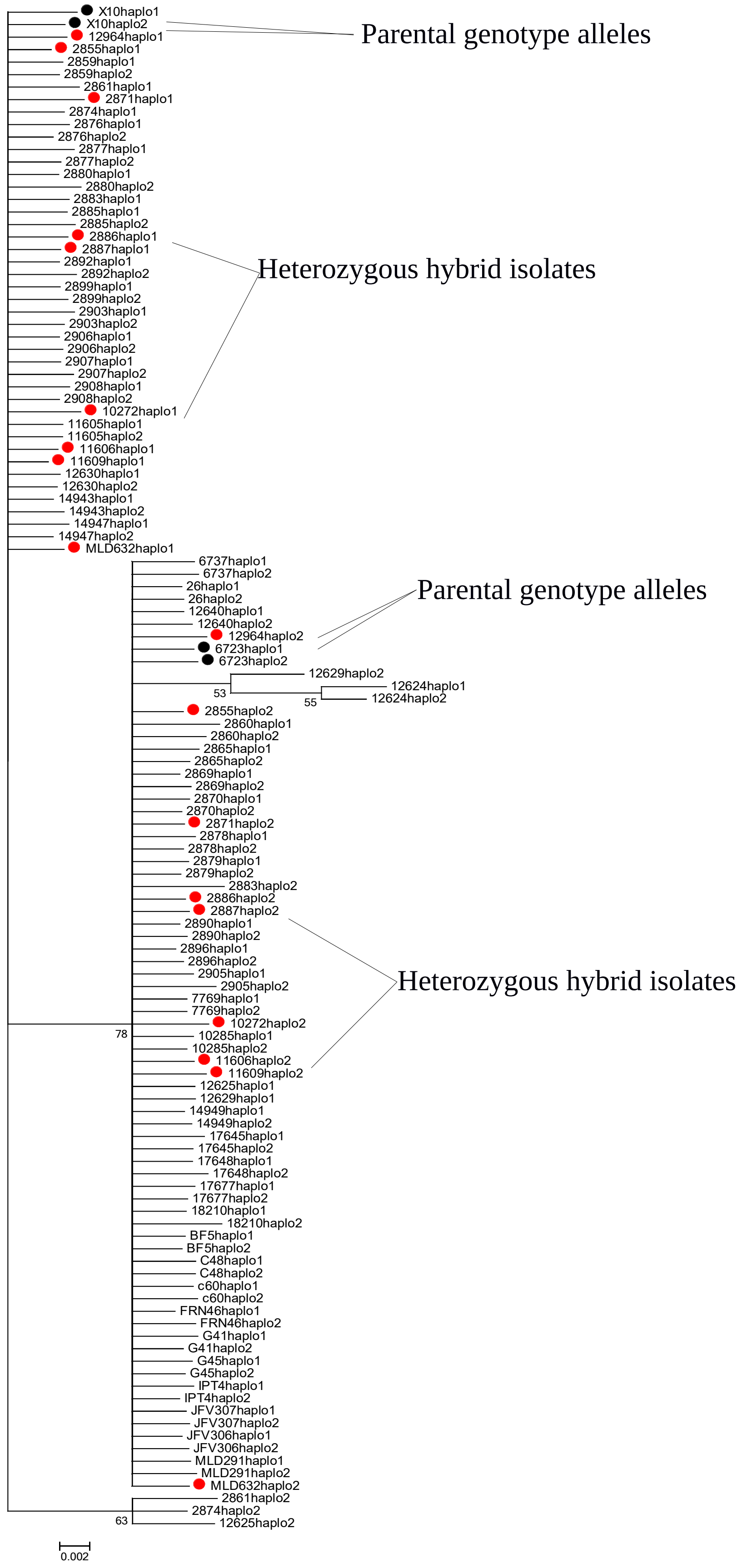

S13 Fig. Haplotypic Bayesian tree with *RB19* locus. Haplotypes inferred by PHASE V2.1. Red circle indicate heterozygous hybrid isolates and black circle correspond to potential parental alleles.
