# Supplementary material for: Dissecting the phyloepidemiology of Trypanosoma cruzi I (TcI) in Brazil by the use of high resolution genetic markers
Source: PLoS Negl Trop Dis. 2018 May 21;12(5):e0006466. doi: 10.1371/journal.pntd.0006466 (PMC5983858; doi:10.1371/journal.pntd.0006466)
Supplement: S18 Fig — (PDF) [file pntd.0006466.s018.pdf]

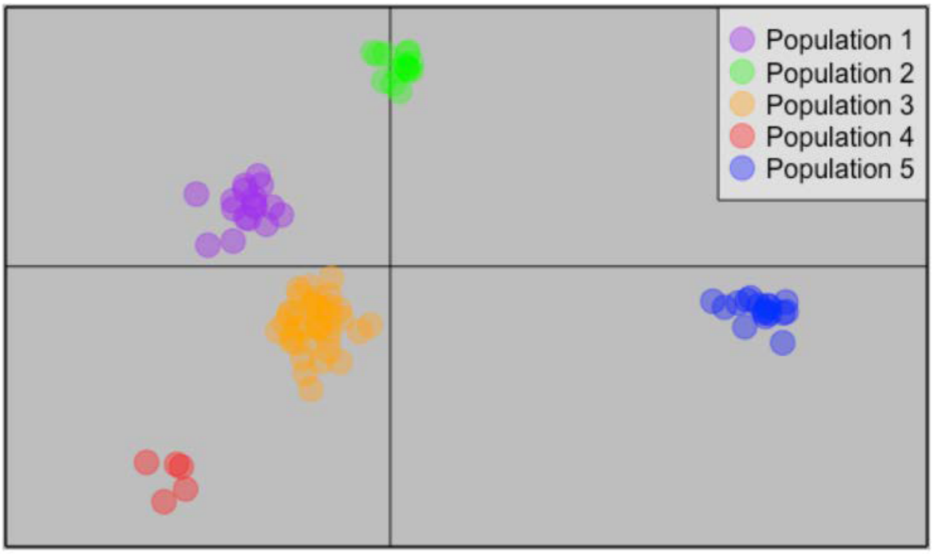

S18 Fig. Nuclear genetic clustering among 92 Brazilian sylvatic TcI strains. Multidimensional scaling plot based on discriminant analysis of principal component (DAPC) analysis for five population clusters defined via *K*-means clustering algorithm (10^9 iterations, over five independent runs, retaining 22 principal components, representing 80% of total variation in the dataset). Individual isolates are represented by dots
