# Supplementary material for: Dissecting the phyloepidemiology of Trypanosoma cruzi I (TcI) in Brazil by the use of high resolution genetic markers
Source: PLoS Negl Trop Dis. 2018 May 21;12(5):e0006466. doi: 10.1371/journal.pntd.0006466 (PMC5983858; doi:10.1371/journal.pntd.0006466)
Supplement: S19 Fig — Comparation between (A) MLST and (B) MLMT trees. (PDF) [file pntd.0006466.s019.pdf]

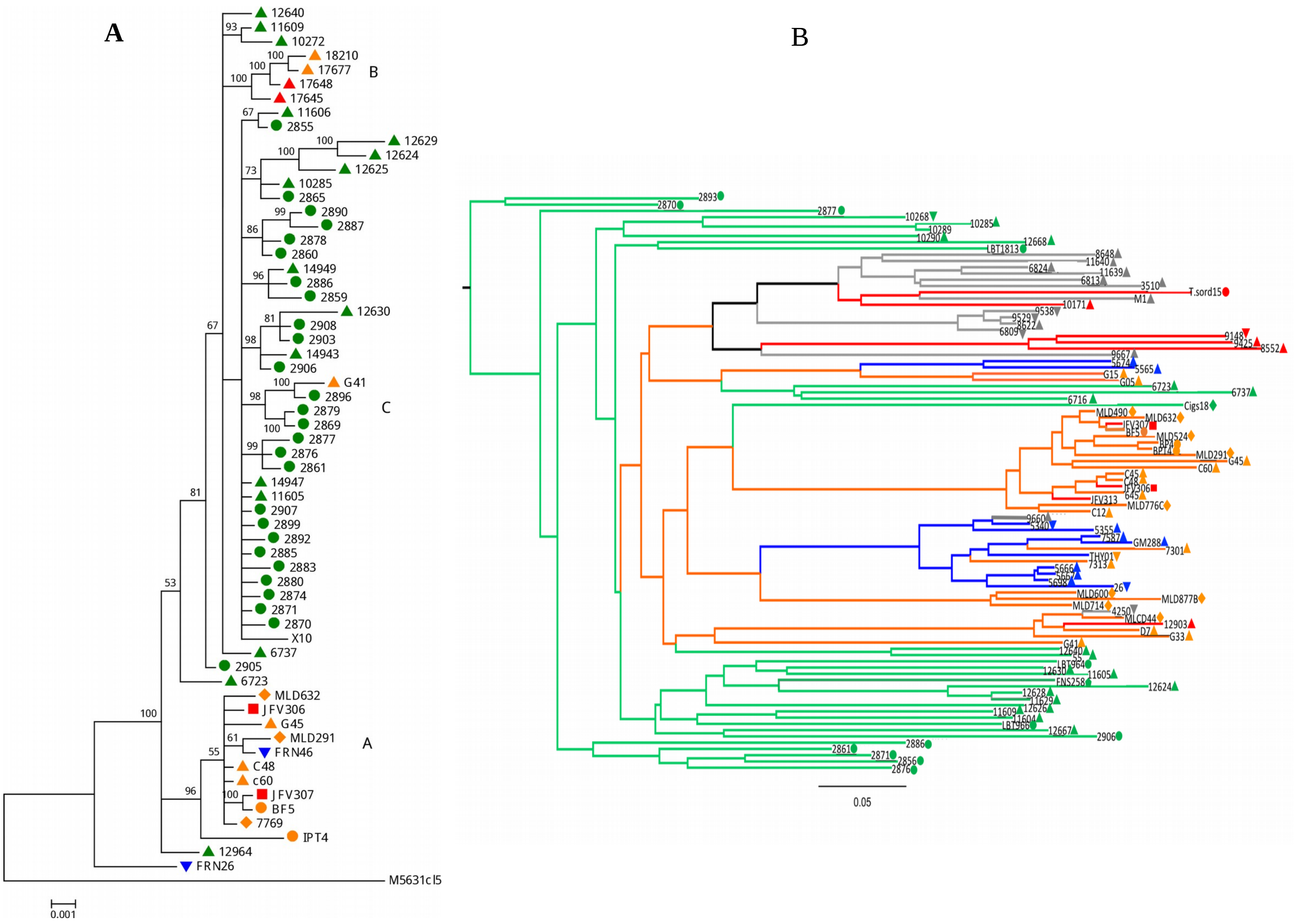

S19 Fig. Comparation between (A) MLST and (B) MLMT trees. Biomes are colors coded; red, Cerrado; greed, Amazon; orange, Atlantic Forest; blue, Pantanal; gray, Caatinga.
