# Supplementary material for: Dissecting the phyloepidemiology of Trypanosoma cruzi I (TcI) in Brazil by the use of high resolution genetic markers
Source: PLoS Negl Trop Dis. 2018 May 21;12(5):e0006466. doi: 10.1371/journal.pntd.0006466 (PMC5983858; doi:10.1371/journal.pntd.0006466)
Supplement: S1 Table — (PDF) [file pntd.0006466.s021.pdf]

S1 Table. *Trypanosoma cruzi* I isolates used in the study

Isolates	Host/vector	Municipality/State	Biome	Year	Latitude	Longitude	MLST	Maxicircle (COII)	MLMT	Genetic cluster based on DAPC
**G41**	*Didelphis marsupialis*	Guapimirim/RJ	Atlantic Forest	2003	-22.532	-42.990	X	X	X	3
**17677**	*Didelphis aurita*	Rio de Janeiro/RJ	Atlantic Forest	2012	-22.563	-43.242	X	X		
**MLD 291**	*Leontopithecus rosalia*	Silva Jardim/RJ	Atlantic Forest	1996	-22.659	-42.383	X	X	X	3
**G45**	*Didelphis aurita*	Silva Jardim/RJ	Atlantic Forest	2000	-22.659	-42.383	X	X	X	3
**MLD 776 (7769)**	*Leontopithecus rosalia*	Silva Jardim/RJ	Atlantic Forest	2001	-22.659	-42.383	X	X	X	5
**C60**	*Philander frenatus*	Teresópolis/RJ	Atlantic Forest	2009	-22.399	-43.011	X	X	X	5
**2877**	*Rhodnius pictipes*	Belem/PA	Amazon	2011	-1.499	-48.490	X	X	X	3
**BF5**	*Rhodnius prolixus*	Teresópolis/RJ	Atlantic Forest	1995	-22.399	-43.011	X	X	X	5
**MLD 632**	*Leontopithecus rosalia*	Silva Jardim/RJ	Atlantic Forest	1996	-22.659	-42.383	X	X	X	5
**C48**	*Philander frenatus*	Teresópolis/RJ	Atlantic Forest	1994	-22.400	-43.011	X		X	5
**2890**	*Rhodnius* sp	Belem/PA	Amazon	2011	-1.499	-48.491	X	X		
**18210**	*Didelphis aurita*	Rio de Janeiro/RJ	Atlantic Forest	2012	-22.940	-43.403	X	X		
**IPT4**	*Rhodnius prolixus*	Teresópolis/RJ	Atlantic Forest	1992/1994	-22.400	-43.011	X		X	3
**2908**	*Rhodnius robustus*	Belem/PA	Amazon	2011	-1.488	-48.438	X			
**JFV 307**	*Phyllostomus albicola*	Arraias/TO	Cerrado	2008	-12.926	-46.935	X	X	X	2
**JFV 306**	*Carolia perspicillata*	Arraias/TO	Cerrado	2008	-12.926	-46.935	X	X	X	5
**17648**	*Didelphis marsupialis*	Ananas/TO	Cerrado	2012	-6.405	-48.095	X	X		
**17645**	*Didelphis* sp	Ananas/TO	Cerrado	2012	-6.405	-48.095	X	X		
**FRN 26\26**	*Oecomys mamorae*	Aquidauana/MG	Pantanal	2002	-19.575	-56.246	X	X	X	2
**FRN 46**	*Oecomys mamorae*	Aquidauana/MG	Pantanal	2002	-19.582	-56.246	X	X		
**2903**	*Rhodnius pictipes*	Belem/PA	Amazon	2011	-1.488	-48.438	X	X		
**2892**	*Rhodnius* sp	Belem/PA	Amazon	2011	-1.500	-48.491	X	X		
**2878**	*Rhodnius pictipes*	Belem/PA	Amazon	2011	-1.500	-48.491	X	X		
**2870**	*Rhodnius pictipes*	Belem/PA	Amazon	2011	-1.500	-48.491	X	X	X	4
**2907**	*Rhodnius robutus*	Belem/PA	Amazon	2011	-1.488	-48.438	X	X		
**2874**	*Rhodnius pictipes*	Belem/PA	Amazon	2011	-1.499	-48.490	X	X		
**12640 (55)**	*Didelphis marsupialis*	Abaetetuba/PA	Amazon	2008	-1.730	-48.872	X	X	X	3
**2885**	*Rhodnius* sp	Belem/PA	Amazon	2011	-1.499	-48.490	X	X		
**2886**	*Rhodnius* sp	Belem/PA	Amazon	2011	-1.499	-48.490	X	X	X	3
**2880**	*Rhodnius* sp	Belem/PA	Amazon	2011	-1.499	-48.490	X	X		
**2905**	*Rhodnius pictipes*	Belem/PA	Amazon	2011	-1.504	-48.449	X	X		
**2859**	*Rhodnius pictipes*	Belem/PA	Amazon	2011	-1.499	-48.490	X			
**14947**	*Philander opossum*	Belem/PA	Amazon	2011	-1.491	-48.451	X	X		
**2861**	*Rhodnius pictipes*	Belem/PA	Amazon	2011	-1.499	-48.490	X	X	X	3
**11609**	*Philander opossum*	Abaetetuba/PA	Amazon	2008	-1.718	-48.883	X	X	X	3
**2906**	*Rhodnius pictipes*	Belem/PA	Amazon	2011	-1.488	-48.438	X	X	X	3
**2896**	*Rhodnius robusto*	Belem/PA	Amazon	2011	-1.499	-48.490	X	X		
**2883**	*Rhodnius* sp	Belem/PA	Amazon	2011	-1.499	-48.490	X	X		
**2899**	*Rhodnius robusto*	Belem/PA	Amazon	2011	-1.499	-48.490	X	X		
**109 (LBCE12624)**	*Philander opossum*	Abaetetuba/PA	Amazon	2008	-1.776	-48.893	X		X	3
**2865**	*Rhodnius pictipes*	Belem/PA	Amazon	2011	-1.499	-48.490	X	X		
**11606**	*Didelphis marsupialis*	Abaetetuba/PA	Amazon	2008	-1.718	-48.883	X	X		
**2879**	*Rhodnius pictipes*	Belem/PA	Amazon	2011	-1.499	-48.490	X	X		
**2860**	*Rhodnius* sp	Belem/PA	Amazon	2011	-1.499	-48.490	X			
**2887**	*Rhodnius* sp	Belem/PA	Amazon	2011	-1.501	-48.460	X	X		
**14943**	*Philander opossum*	Belem/PA	Amazon	2011	-1.499	-48.490	X	X		
**2871**	*Rhodnius pictipes*	Belem/PA	Amazon	2011	-1.492	-48.451	X	X	X	3
**12630**	*Philander opossum*	Abaetetuba/PA	Amazon	2008	-1.730	-48.872	X	X	X	3
**88 (LBCE 6723)**	*Didelphis marsupialis*	Itupiranga/PA	Amazon	2004	-5.173	-49.366	X		X	3
**10285**	*Didelphis marsupialis*	Cachoeira do Arari/PA	Amazon	2006	-1.004	-48.957	X	X	X	3
**11605**	*Philander opossum*	Abaetetuba/PA	Amazon	2008	-1.730	-48.872	X	X	X	3
**14949**	*Philander opossum*	Belem/PA	Amazon	2011	-1.489	-48.437	X	X		
**2876**	*Rhodnius pictipes*	Belem/PA	Amazon	2011	-1.492	-48.451	X	X	X	3
**2855**	*Rhodnius pictipes*	Belem/PA	Amazon	2011	-1.492	-48.451	X	X		
**2869**	*Rhodnius pictipes*	Belem/PA	Amazon	2011	-1.492	-48.451	X	X		
**10272**	*Didelphis marsupialis*	Cachoeira do Arari/PA	Amazon	2006	-1.004	-48.957	X	X		
**70 (LBCE 12964)**	*Didelphis marsupialis*	Curralinho/PA	Amazon	2009	-0.536	-49.184	X			
**12629**	*Philander opossum*	Abaetetuba/PA	Amazon	2008	-1.758	-49.057	X	X		
**2 (LBCE 6737)**	*Didelphis marsupialis*	Itupiranga/PA	Amazon	2004	-5.173	-49.366	X		X	3
**12625**	*Didelphis marsupialis*	Abaetetuba/PA	Amazon	2008	-1.730	-48.872	X	X		
**23 (6716)**	*Didelphis marsupialis*	Itupiranga/PA	Amazon	2004	-5.173	-49.366			X	3
**645**	*Didelphis marsupialis*	Teresópolis/RJ	Atlantic Forest	1992	-22.399	-43.011			X	5

**2856**	*Rhodnius pictipes*	Belem/PA	Amazon	2011	-1.491	-48.451			X	3
**2868**	*Rhodnius pictipes*	Belem/PA	Amazon	2011	-1.491	-48.451		X		
**2884**	*Rhodnius* sp	Belem/PA	Amazon	2011	-1.491	-48.451		X		
**2902**	*Rhodnius pictipes*	Belem/PA	Amazon	2011	-1.489	-48.437		X		
**2904**	*Rhodnius pictipes*	Belem/PA	Amazon	2011	-1.489	-48.437				
**7313**	*Didelphis aurita*	Navegantes/SC	Atlantic Forest	2005	-26.899	-48.656			X	5
**10290**	*Didelphis marsupialis*	Cachoeira do Arari/PA	Amazon	2006	-1.004	-48.957		X	X	
**C12**	*Philander frenatus*	Teresópolis/RJ	Atlantic Forest	1992/1994	-22.399	-43.011		X	X	3
**C45**	*Philander frenatus*	Teresópolis/RJ	Atlantic Forest	1992/1994	-22.399	-43.011		X	X	2
**G15**	*Didelphis marsupialis*	Silva Jardim/RJ	Atlantic Forest	2003	-22.532	-42.990			X	5
**JFV 313**	*Phyllostomus hastatus*	Arraias/TO	Cerrado	2008	-12.926	-46.935		X	X	5
**12628**	*Didelphis marsupialis*	Abaetetuba/PA	Amazon	2008	-1.730	-48.872			X	3
**G05**	*Didelphis* sp	Silva Jardim/RJ	Atlantic Forest	2003	-22.532	-42.990			X	5
**Cigs 18**	*Saguinus bicolor*	Manaus/AM	Amazon	2000	-3.100	-60.045			X	3
**2893**	*Rhodnius robusto*	Belem/PA	Amazon	2011	-1.492	-48.450			X	3
**9660**	*Monodelphis domestica*	Redencao/CE	Caatinga	2006	-4.179	-38.730			X	2
**12667***	*Didelphis marsupialis*	Curralinho/PA	Amazon	2009	-0.536	-49.184			X	3
**10268***	*Proechimys sp*	Cachoeira do Arari/PA	Amazon	2006	-1.004	-48.957			X	3
**10171***	*Didelphis albiventris*	São Raimundo Nonato/PI	Cerrado	2006	-9.967	-45.717		X	X	1
**10289***	*Oryzomys sp.*	Cachoeira do Arari/PA	Amazon	2006	-1.004	-48.957			X	3
**12668***	*Didelphis marsupialis*	Curralinho/PA	Amazon	2009	-0.536	-49.184			X	3
**LBT1813***	*Rhodnius pictipes*	Belém/PA	Amazon	2009	-1.379	-48.476			X	3
**8648***	*Didelphis albiventris*	Jaguaruana/CE	Caatinga	2005	-4.831	-37.781			X	1
**11640***	*Didelphis albiventris*	Russas/Ceará	Caatinga	2008	-4.939	-37.979			X	1
**6824***	*Didelphis albiventris*	Jaguaruana/CE	Caatinga	2004	-4.831	-37.781		X	X	1
**11639***	*Didelphis albiventris*	Russas/CE	Caatinga	2008	-4.939	-37.979			X	1
**6813***	*Didelphis albiventris*	Jaguaruana/CE	Caatinga	2004	-4.831	-37.781			X	1
**3510***	*Didelphis albiventris*	Jaguaruana/CE	Caatinga	2001	-4.831	-37.781			X	1
**T sord***	*Triatoma sordida*	Posse/GO	Cerrado	2008	-14.086	-46.371			X	4
**M1***	*Didelphis albiventris*	Coronel José Dias/PI	Caatinga	1998	-8.825	-42.506			X	4
**9538***	*Rattus rattus*	Jaguaruana/CE	Caatinga	2006	-4.831	-37.781			X	1
**9529***	*Rattus rattus*	Jaguaruana/CE	Caatinga	2006	-4.831	-37.781			X	1
**8622***	*Didelphis albiventris*	Jaguaruana/CE	Caatinga	2005	-4.831	-37.781			X	1
**6809***	*Rattus rattus*	Jaguaruana/CE	Caatinga	2004	-4.831	-37.781			X	1
**9148***	*Gracilinanus sp*	Aporé/GO	Cerrado	2006	-18.949	-51.909			X	1
**9425***	*Didelphis albiventris*	Aporé/GO	Cerrado	2006	-18.949	-51.909			X	1
**8552***	*Didelphis albiventris*	Aporé/GO	Cerrado	2005	-18.949	-51.909			X	5
**9667***	*Monodelphis domestica*	Redenção/CE	Caatinga	2006	-4.226	-38.731		X	X	1
**5674***	*Monodelphis domestica*	Corumbá/MS	Pantanal	2003	-19.010	-57.655			X	2
**5565***	*Didelphis aurita*	Capitão Andrade/MG	Atlantic Forest	2003	-19.070	-41.863			X	3
**MLD490***	*Leontopithecus rosalia*	Silva Jardim/RJ	Atlantic Forest	1996	-22.659	-42.383			X	4
**MLD524***	*Leontopithecus rosalia*	Silva Jardim/RJ	Atlantic Forest	1996	-22.659	-42.383			X	5
**BP4***	*Rhodnius prolixus*	Teresópolis/RJ	Atlantic Forest	1992/1994	-22.399	-43.011			X	3
**7301***	*Didelphis aurita*	Navegantes/SC	Atlantic Forest	2005	-26.899	-48.656			X	2
**THY01***	*Thylamys macrurus*	Corumbá/MS	Pantanal	2005	-18.992	-56.631			X	2
**MLD600***	*Leontopithecus rosalia*	Silva Jardim/RJ	Atlantic Forest	1999	-22.659	-42.383			X	5
**MLD877***	*Leontopithecus rosalia*	Silva Jardim/RJ	Atlantic Forest	2003	-22.659	-42.383		X	X	5
**MLD714***	*Leontopithecus rosalia*	Silva Jardim/RJ	Atlantic Forest	1998	-22.659	-42.383			X	2
**4250***	*Thrichomys apereoides*	São Raimundo Nonato/PI	Caatinga	2001	-9.005	-45.711			X	1
**MLCD44***	*Leontopithecus chrysomela*	Ilhéus/BA	Atlantic Forest	2003	-15.269	-39.067			X	4
**12903***	*Gracilinanus sp.*	Dianópolis/TO	Cerrado	2009	-11.628	-46.821			X	1
**D7***	*Didelphis aurita*	Silva Jardim/RJ	Atlantic Forest	1996	-22.659	-42.383			X	5
**G33***	*Didelphis marsupialis*	Silva Jardim/RJ	Atlantic Forest	2000	-22.532	-42.990			X	5
**LBT964***	*Rhodnius pictipes*	Abaetetuba/PA	Amazon	2011	-0.536	-49.184			X	3
**11629***	*Philander opposum*	Abaetetuba/PA	Amazon	2008	-1.730	-48.872			X	1
**12626***	*Micoureus demerarae*	Abaetetuba/PA	Amazon	2008	-1.730	-48.872			X	3
**11604***	*Marmosops murina*	Abaetetuba/PA	Amazon	2008	-1.730	-48.872			X	3
**LBT966***	*Monodelphis domestica*	Redencao/CE	Caatinga	2008	-4.939	-37.979			X	1
**5340***	*Oecomys sp*	Aquidauana/MS	Pantanal	2003	-19.681	-57.338			X	2
**5355***	*Monodelphis domestica*	Aquidauana/MS	Pantanal	2003	-19.139	-56.796			X	2
**7587***	*Gracilinanus agilis*	Corumbá/MS	Pantanal	2005	-19.010	-57.655			X	2
**GM288***	*Nasua nasua*	Corumbá/MS	Pantanal	2007	-19.010	-57.655			X	2
**5666***	*Gracilinanus agilis*	Corumbá/MS	Pantanal	2003	-19.139	-56.796			X	2
**5667***	*Gracilinanus agilis*	Corumbá/MS	Pantanal	2003	-19.139	-56.796			X	2
**5698***	*Gracilinanus sp*	Corumbá/MS	Pantanal	2004	-19.139	-56.796			X	3
**FNS258***	*Canis familiaris*	Abaetetuba/PA	Amazon	2008	-1.730	-48.872			X	3

**T. cruzi* I isolates previously published (Lima *et al.* 2014).

MS, Mato Grosso o Sul; **CE**, Ceara; **PA**, Para; **RJ**, Rio de Janeiro; **TO**, Tocantins; **BA**, Bahia; **PI**, Piaui; **SC**, Santa Catarina; **MG**, Minas Gerais; **AM**, Amazonas
