# Supplementary material for: Dissecting the phyloepidemiology of Trypanosoma cruzi I (TcI) in Brazil by the use of high resolution genetic markers
Source: PLoS Negl Trop Dis. 2018 May 21;12(5):e0006466. doi: 10.1371/journal.pntd.0006466 (PMC5983858; doi:10.1371/journal.pntd.0006466)
Supplement: S2 Table — (PDF) [file pntd.0006466.s022.pdf]

S2 Table. MLST gene targets

Gene ID	Chromosome number	Primer sequence (5'3')	Amplicon size (bp)	Fragment length (bp)
CoAR	32	AGGAGGCTTTTGAGTCCACA (20)	554	514
		TCCAACAACACCAACCTCAA (20)		
GTP	12	TGTGACGGGACATTTTACGA (20)	561	521
		CCCCTCGATCTCACGATTTA (20)		
LAP	27	TGTACATGTTGCTTGGCTGAG (21)	444	402
		GCTGAGGTGATTAGCGACAAA (21)		
PDH	40	GGGGCAAGTGTTTGAAGCTA (20)	491	451
		AGAGCTCGCTTCGAGGTGTA (20		
Rb19	29	GCCTACACCGAGGAGTACCA (20)	408	340
		TTCTCCAATCCCCAGACTTG (20)		
RHO1	8	AGTTGCTGCTTCCCATCAAT (20)	455	415
		CTGCACAGTGTATGCCTGCT (20)		
