# Supplementary material for: Dissecting the phyloepidemiology of Trypanosoma cruzi I (TcI) in Brazil by the use of high resolution genetic markers
Source: PLoS Negl Trop Dis. 2018 May 21;12(5):e0006466. doi: 10.1371/journal.pntd.0006466 (PMC5983858; doi:10.1371/journal.pntd.0006466)
Supplement: S3 Table — (PDF) [file pntd.0006466.s023.pdf]

S3 Table. Panel of microsatellite loci and primers.

Chromosome*	Primer code	Repeat type	Forward/Reverse Primer (5'-3')
**6**	6529(CA)$_a$	(CA)$_n$	TGTGAAATGATTTGACCCGA
			AGAGTCACGCCGCAAAGTAT
**10**	6855(TA)(GA)	(TA)$_n$(GA)$_n$	TGTGATCAACGCGCATAAAT
			TTCCATTGCCTCGTTTTAGA
**15**	11863(CA)	(CA)$_n$	AGTTGACATCCCCAAGCAAG
			CCCTGATGCTGCAGACTCTT
**19**	TcUn3	Unknown	CTTAAAGAGATACAAGAGGGAAGG
			CTGTTATTTCAATAACACGGGG
**19**	10101(TA)	(TA)$_n$	AACCCGCGCAGATACATTAG
			TTCATTTGCAGCAACACACA
**24**	8741(TA)	(TA)$_n$	TGTAACGGTAGGTCTCAATTCG
			TTGCACTTGTGTATCTCGCC
**27**	10101(TC)	(TC)$_n$	CGTACGACGTGGACACAAAC
			ACAAGTGGGTGAGCCAAAAG
**27**	10101(CA)$_c$	(CA)$_n$	GTGTCGTTGCTCCCAAACTC
			AAACTTGCCAAATGTGAGGG
**27**	10101(CA)$_a$	(CA)$_n$	GTCGCCATCATGTACAAACG
			CTGTTGGCGAATGGTCATAA
**34**	6559(TC)	(TC)$_n$	CGCTCTCAAAGGCACCTTAC
			ATATGGACGCGTAGGAGTGC
**37**	10187(TTA)	(TTA)$_n$	GAGAGAGATTCGGAAACTAATAGC
			CATGTCCCTTCCTCCGTAAA
**37**	10187(CA)(TA)	(CA)$_n$(TA)$_n$	CATGTCATTAAGTGGCCACG

			GCACATGTTGGTTGTTGGAA
**37**	10187(GA)	$(GA)_n$	GTCACACCACTAGCGATGACA
			ACTGCACAATACCCCCTTTG
**37**	TcUn2	Unknown	AACAAAATCTAGCGTCTACCATCC
			GGTGTTGGCGTGTATGATTG
**37**	TcUn4	Unknown	ATGCTCCGCAACATATTACTCA
			GTCGAGCTTCTGTTGTTCCC
**39**	$6925(TG)_b$	$(TG)_n$	GAAACGCACTCACCCACAC
			GGTAGCAACGCCAAACTTTC
**39**	6925(CT)	$(CT)_n$	CATCAAGGAAAAACGGAGGA
			CGGTACCACCTCAAGGAAAG
**39**	7093(TC)	$(TC)_n$	CCAACATTCAACAAGGGAAA
			GCATGAATATTGCCGGATCT
**39**	$7093(TA)_c$	$(TA)_n$	CGTGTGCACAGGAGAGAAAA
			CGTTTGGAGGAGGATTGAGA
**39**	7093(TA)b	(TA)n	GGAAACACATCACGCAAAGA
			CTCTCATCTTTTGTTGTGTCCG
**39**	$6925(TG)_a$	$(TG)_n$	TCGTTCTCTTTACGCTTGCA
			TAGCAGCACCAAACAAAACG
**39**	7093(TCC)	$(TCC)_n$	AGACGTTCATATTCGCAGCC
			AGCCACATCCACATTTCCTC
**40**	11283(TCG)	$(TCG)_n$	ACCACCAGGAGGACATGAAG
			TGTACACGGAACAGCGAAG
**40**	$11283(TA)_b$	$(TA)_n$	AACATCCTCCACCTCACAGG

			TTTGAATGCGAGGTGGTACA
**41**	10359 (CA)(GA)	$(CA)_n(GA)_n$	AGTCCTACTGCCTCCTTGCA
			CTGTTGGCGAATGGTCATAA
