# Supplementary material for: Dissecting the phyloepidemiology of Trypanosoma cruzi I (TcI) in Brazil by the use of high resolution genetic markers
Source: PLoS Negl Trop Dis. 2018 May 21;12(5):e0006466. doi: 10.1371/journal.pntd.0006466 (PMC5983858; doi:10.1371/journal.pntd.0006466)
Supplement: S4 Table — (PDF) [file pntd.0006466.s024.pdf]

S4 Table. SNP data of isolates for *RB19* with highlighted (gray) putative donors (D) and recipient (R) isolates. SNPs that did not appear in different clusters are highlighted in yellow.

Isolate	1	2	3	4
D X10	C	G	A	G
6737	C	G	G	G
26	T	G	G	G
12640	C	G	G	G
R 12964	C	G	R	G
D 6723	C	G	G	G
12624	T	C	G	G
R 2855	C	G	R	G
2859	C	G	A	G
2860	C	G	G	G
2861	C	S	A	G
2865	C	G	G	G
2869	C	G	G	G
2870	C	G	G	G
R 2871	C	G	R	G
2874	C	S	A	G
2876	C	G	A	G
2877	C	G	A	G
2878	C	G	G	G
2879	C	G	G	G
2880	C	G	A	G
2883	C	S	R	G
2885	C	G	A	G
R 2886	C	G	R	G
R 2887	C	G	R	G
2890	C	G	G	G
2892	C	G	A	G
2896	C	G	G	G
2899	C	G	A	G
2903	C	G	A	G
2905	C	G	G	G
2906	C	G	A	G
2907	C	G	A	G
2908	C	G	A	G
7769	T	G	G	G
R 10272	C	G	R	G
10285	C	G	G	G
11605	C	G	A	G
R 11606	C	G	R	G
R 11609	C	G	R	G
12625	C	S	R	G
12629	T	S	G	G
12630	C	G	A	G
14943	C	G	A	G
14947	C	G	A	G
14949	C	G	G	G
17645	C	G	G	R
17648	C	G	G	R
17677	C	G	G	R

**18210**	C	G	G	R
**BF5**	T	G	G	G
**C48**	T	G	G	G
**c60**	T	G	G	G
**11D**	C	G	G	G
**FRN46**	T	G	G	G
**G41**	C	G	G	G
**G45**	T	G	G	G
**IPT4**	T	G	G	G
**JFV307**	T	G	G	G
**JFV306**	T	G	G	G
**MLD291**	T	G	G	G
**R MLD632**	C	G	R	G
