# Supplementary material for: Dissecting the phyloepidemiology of Trypanosoma cruzi I (TcI) in Brazil by the use of high resolution genetic markers
Source: PLoS Negl Trop Dis. 2018 May 21;12(5):e0006466. doi: 10.1371/journal.pntd.0006466 (PMC5983858; doi:10.1371/journal.pntd.0006466)
Supplement: S5 Table — (PDF) [file pntd.0006466.s025.pdf]

S5 Table. SNP data of isolates for *PDH* with highlighted (gray) putative donors (D) and recipient (R) isolates. SNPs that did not appear in different clusters are highlighted in yellow.

Isolate	1	2	3	4	5
X10	T	A	C	Y	T
6737	T	A	C	T	T
26	T	A	C	C	C
12640	T	A	C	T	T
12964	T	A	T	Y	Y
6723	T	A	C	Y	Y
12624	T	A	C	T	T
2855	T	A	C	T	T
2859	T	A	C	T	T
2860	T	A	C	T	T
2861	T	A	C	T	T
2865	T	A	C	T	T
D 2869	A	A	C	T	T
2870	T	A	C	T	T
2871	T	A	C	T	T
2874	T	A	C	T	T
2876	T	A	C	T	T
2877	T	A	C	T	T
2878	T	A	C	T	T
D 2879	A	A	C	T	T
2880	T	A	C	T	T
2883	T	A	C	T	T
2885	T	A	C	T	T
2886	T	A	C	T	T
2887	T	A	C	T	T
2890	T	A	C	T	T
R 2892	W	A	C	T	T
R 2896	W	A	C	T	T
2899	T	A	C	T	T
2903	T	A	C	T	T
2905	T	A	C	T	T
2906	T	A	C	T	T
2907	T	A	C	T	T
2908	T	A	C	T	T
7769	T	A	C	T	T
10272	T	A	C	T	T
R 10285	W	A	C	T	T
11605	T	A	C	T	T
11606	T	A	C	T	T
11609	T	A	C	T	T
12625	T	A	C	T	Y
12629	T	A	C	T	T
12630	T	A	C	T	T

**14943**	T	A	C	T	T
**14947**	T	A	C	T	T
**14949**	T	A	C	T	T
**R 17645**	W	A	C	T	T
**17648**	T	A	C	T	T
**17677**	T	A	C	T	T
**18210**	T	A	C	T	T
**BF5**	T	A	C	T	Y
**C60**	T	A	C	T	T
**C48**	T	A	C	T	Y
**FRN46**	T	A	C	T	T
**R G41**	W	A	C	T	T
**G45**	T	T	C	T	T
**IPT4**	T	A	C	T	Y
**JFV 306**	T	A	C	T	C
**JFV 307**	T	A	C	T	Y
**MLD 291**	T	A	C	T	Y
**MLD 632**	T	A	C	T	Y
