# Supplementary material for: Dissecting the phyloepidemiology of Trypanosoma cruzi I (TcI) in Brazil by the use of high resolution genetic markers
Source: PLoS Negl Trop Dis. 2018 May 21;12(5):e0006466. doi: 10.1371/journal.pntd.0006466 (PMC5983858; doi:10.1371/journal.pntd.0006466)
Supplement: S6 Table — (PDF) [file pntd.0006466.s026.pdf]

S6 Table. SNP data of isolates for *GTP* with highlighted (gray) putative donors (D) and recipient (R) isolates. SNPs that did not appear in different clusters are highlighted in yellow.

Strain	1	2	3	4	5	6	7
**X10**	Y	C	T	T	A	A	T
**6737**	C	C	T	T	A	A	T
**26**	C	A	T	G	A	G	C
**12640**	T	C	T	T	A	A	T
**12964**	C	C	T	T	A	A	Y
**6723**	C	C	T	T	A	A	T
**R 12624**	Y	C	T	T	A	A	T
**2855**	C	C	T	T	A	A	T
**2859**	C	C	T	T	A	A	Y
**2860**	C	C	T	T	A	A	T
**2861**	C	C	T	T	A	A	T
**2865**	C	C	T	T	A	A	T
**2869**	C	C	T	T	A	A	T
**2870**	C	C	T	T	A	A	Y
**2871**	C	C	T	T	A	A	T
**2874**	C	C	T	T	A	A	T
**2876**	C	C	T	T	A	A	T
**2877**	C	C	T	T	A	A	T
**2878**	C	C	T	T	A	A	T
**2879**	C	C	T	T	A	A	T
**2880**	C	C	T	T	A	A	T
**2883**	C	C	T	T	A	A	T
**2885**	C	C	T	T	A	A	T
**2886**	C	C	T	T	A	A	Y
**2887**	C	C	T	T	A	A	T
**2890**	C	C	T	T	A	A	T
**2892**	C	C	T	T	A	A	T
**2896**	C	C	T	T	A	A	T
**2899**	C	C	T	T	A	A	T
**2903**	C	C	T	T	A	A	T
**2905**	C	C	T	T	A	A	T
**2906**	C	C	T	T	A	A	T
**2907**	C	C	T	T	A	A	T
**2908**	C	C	T	T	A	A	T
**7769**	C	C	T	T	A	A	Y
**10272**	C	C	Y	T	A	A	Y
**D 10285**	C	C	T	T	A	A	T
**11605**	C	C	T	T	A	A	T
**11606**	C	C	T	T	A	A	T
**11609**	C	C	T	T	A	A	T
**12625**	C	C	T	T	A	A	T
**12629**	C	C	T	T	A	A	T

**R 12630**	Y	C	T	T	A	A	T
**D 14943**	T	C	T	T	A	A	T
**14947**	C	C	T	T	A	A	T
**14949**	C	C	T	T	A	A	Y
**17645**	C	C	T	T	A	A	T
**17648**	C	C	T	T	R	A	T
**17677**	C	C	T	T	G	A	T
**18210**	C	C	T	T	G	A	T
**BF5**	C	M	T	T	A	A	Y
**C48**	C	C	T	T	A	A	Y
**C60**	C	C	T	T	A	A	Y
**FRN46**	C	C	T	T	A	A	Y
**G41**	C	C	T	T	A	A	T
**G45**	C	M	T	T	A	A	Y
**IPT4**	C	C	T	T	A	A	Y
**JFV306**	C	C	T	T	A	A	Y
**JFV307**	C	M	T	T	A	A	Y
**MLD291**	C	C	T	T	A	A	C
**MLD632**	C	C	T	T	A	A	Y
