# Supplementary material for: Dissecting the phyloepidemiology of Trypanosoma cruzi I (TcI) in Brazil by the use of high resolution genetic markers
Source: PLoS Negl Trop Dis. 2018 May 21;12(5):e0006466. doi: 10.1371/journal.pntd.0006466 (PMC5983858; doi:10.1371/journal.pntd.0006466)
Supplement: S7 Table — (PDF) [file pntd.0006466.s027.pdf]

	MS1	MS2	MS3	MS4	MS5	MS6	MS7	MS8	MS9	MS10	MS11	MS12	MS13	MS14	MS15	MS16	MS17	MS18	MS19	MS20	MS21	MS22	MS23	MS24	MS25
2870	104	159	160	111	103	172	183	100	185	147	110	172	173	118	160	154	182	128	140	107	116	147	131	115	169
	104	159	167	115	103	172	185	100	256	151	110	180	173	118	160	154	184	128	140	144	116	149	131	115	169
G45	106	159	167	111	113	172	185	100	251	139	110	176	178	118	160	152	182	128	140	132	116	134	131	133	171
	106	161	169	111	115	172	196	100	259	147	110	176	178	118	160	152	186	128	140	135	116	134	131	133	171
C60	104	159	160	105	113	172	185	100	251	139	110	176	178	118	160	152	182	128	140	132	116	136	131	115	171
	106	163	167	111	115	172	196	100	259	147	110	176	178	118	160	152	186	128	140	135	134	136	131	133	171
55	104	159	167	109	103	172	179	100	259	143	110	172	170	118	160	150	182	128	140	107	108	117	131	115	169
	104	163	169	115	105	172	183	100	259	145	110	172	170	118	160	160	188	128	140	132	136	154	131	133	169
2877	104	159	160	115	103	172	183	100	256	145	110	172	166	118	160	152	188	128	140	107	108	117	131	115	169
	104	159	160	115	103	172	183	100	259	151	110	184	176	118	160	154	188	128	140	138	116	149	131	115	169
2886	104	159	160	109	103	172	183	100	255	151	110	180	166	118	160	154	182	128	140	107	108	154	131	115	169
	104	163	174	115	109	172	185	100	259	151	110	180	166	118	160	154	188	128	140	144	143	156	131	133	169
2906	106	159	160	105	103	172	183	100	256	145	110	182	168	118	160	154	184	128	127	132	116	117	134	115	169
	106	159	167	105	109	172	187	100	259	151	110	182	172	118	160	154	184	128	140	138	138	149	134	133	169
2856	104	159	172	115	103	172	183	100	189	147	110	180	166	118	160	154	188	128	140	132	132	152	131	115	169
	104	159	172	115	109	172	188	100	255	151	110	180	166	118	160	154	188	128	140	144	143	156	131	115	169
2876	106	159	158	111	103	172	183	100	255	145	110	180	166	118	160	154	188	128	140	132	116	147	131	115	169
	106	159	172	115	109	172	188	109	255	151	110	180	166	118	160	160	188	128	150	138	116	156	131	115	169
26	104	159	167	105	107	168	183	100	197	143	110	172	175	118	160	154	188	128	140	132	116	117	131	115	169
	104	159	177	105	111	172	198	100	275	147	110	172	178	118	160	154	188	128	140	132	132	145	131	115	169
2861	104	159	160	105	103	172	183	100	255	147	110	182	166	118	160	154	188	128	140	107	116	117	131	115	169
	104	159	172	105	109	172	188	100	255	151	110	182	166	118	160	160	188	128	140	132	116	147	131	115	169
2871	104	159	172	105	103	172	185	100	255	147	110	180	166	118	160	154	188	128	127	132	116	147	131	115	169
	104	159	172	115	109	172	188	100	255	151	110	180	166	118	160	154	188	128	150	144	116	156	131	115	169
2893	104	159	160	111	103	172	183	100	256	147	110	170	173	118	160	154	182	128	127	132	116	117	131	115	169
	104	159	169	115	103	172	183	100	256	151	110	178	175	118	160	160	184	128	140	144	116	147	131	115	169
MLD600	104	159	172	111	107	172	183	100	ND	147	110	168	178	118	160	158	190	128	140	132	116	136	131	115	169
	104	159	176	111	107	172	187	100	285	147	110	168	184	118	160	162	190	128	140	135	116	147	131	115	169
THY01	104	159	167	109	107	167	183	100	273	143	110	172	175	118	160	154	186	128	140	132	116	145	131	115	169
	104	159	177	111	111	170	198	100	275	147	110	172	178	118	160	162	186	128	140	132	116	147	131	115	169
MLD776C	104	159	167	111	113	172	185	100	251	139	110	174	178	118	160	150	182	128	140	132	116	136	131	115	171
	106	159	169	111	115	172	196	100	259	147	110	174	178	118	160	150	186	128	140	135	116	136	131	115	171
MLD632	104	157	167	111	113	172	185	100	251	139	110	176	178	118	160	152	182	128	140	132	116	136	131	115	171
	106	161	169	111	115	172	196	100	259	147	110	176	178	118	160	152	186	128	140	135	116	136	131	115	171
5698	104	159	167	109	107	168	183	100	273	143	110	172	175	118	160	154	188	128	140	132	116	145	131	115	169
	104	159	177	111	111	172	198	100	275	147	110	172	178	118	160	154	188	128	140	132	116	147	131	115	169
645	104	159	167	111	113	172	185	100	251	139	110	174	178	118	160	152	182	128	140	132	116	136	131	115	173
	106	163	169	111	115	172	196	100	259	147	110	174	178	118	160	162	186	128	140	135	116	136	131	115	173
MLD714	104	157	172	111	107	172	183	100	ND	147	110	172	178	118	160	158	190	128	140	132	116	136	131	115	169

	104	159	176	111	107	172	187	100	285	147	110	172	181	118	160	158	190	128	140	135	116	147	131	115	169
D7	106	157	166	111	103	172	183	100	255	143	110	180	170	118	160	156	184	125	138	107	116	142	131	115	169
	106	163	169	111	105	172	183	100	259	147	110	180	170	118	160	160	188	128	142	135	134	142	131	115	169
9425	104	161	172	109	109	172	182	100	251	137	106	170	166	118	160	152	192	128	136	141	116	154	131	115	163
	104	164	172	109	113	172	187	100	ND	143	106	178	166	118	160	156	192	128	142	144	116	156	131	115	169
BF5	104	159	167	111	113	172	185	100	251	139	110	176	178	118	160	152	182	128	140	132	116	136	131	115	171
	106	161	169	111	115	172	196	100	259	147	110	176	178	118	160	160	186	128	140	135	116	136	131	115	171
7587	104	157	167	109	107	168	183	100	273	143	110	172	175	118	160	154	188	128	140	132	116	145	131	115	169
	104	157	177	109	111	172	198	100	273	147	110	172	178	118	160	154	188	128	140	132	116	147	131	115	169
BP4	104	157	167	111	113	172	185	100	251	139	110	174	178	118	160	152	182	128	140	132	116	136	131	115	171
	106	157	169	111	113	172	196	100	259	147	110	174	178	118	160	152	186	128	140	135	116	136	131	115	171
10289	104	155	174	109	103	170	183	100	256	141	110	180	176	118	160	154	184	128	140	107	116	147	131	112	169
	104	164	174	115	109	172	188	100	259	151	110	182	178	118	160	154	188	128	144	132	116	149	131	115	169
BPT4	104	157	167	111	113	172	185	100	251	139	110	174	178	118	160	152	182	128	140	132	116	136	131	115	171
	106	157	169	111	115	172	196	100	259	147	110	174	178	118	160	152	186	128	140	135	116	136	131	115	171
MLD877B	104	159	172	111	107	172	183	100	251	147	110	166	178	127	160	158	190	128	140	132	116	136	131	115	169
	104	159	176	111	107	172	187	100	ND	147	110	172	181	127	166	162	190	128	140	135	116	147	131	115	169
G33	106	157	166	111	103	170	183	100	255	143	110	178	169	118	160	156	184	125	138	107	116	142	131	115	169
	106	163	169	111	105	170	183	100	259	147	110	178	169	118	160	158	188	128	142	135	116	142	131	115	169
7301	104	157	167	109	107	167	183	100	273	143	110	172	176	118	160	154	186	128	140	132	116	145	131	115	169
	104	157	177	109	111	170	198	100	275	147	110	172	178	118	160	154	186	128	140	132	130	147	131	115	169
8552	104	161	172	109	109	170	181	100	251	137	106	170	166	118	160	156	190	128	136	107	116	122	131	115	163
	104	164	172	111	113	170	187	100	ND	151	108	174	166	118	160	156	190	128	136	141	116	154	131	115	169
G05	104	161	169	109	107	172	183	100	255	143	110	180	175	118	160	158	182	128	144	129	116	136	131	115	169
	106	159	169	111	107	172	187	100	255	153	110	180	175	118	160	158	182	128	144	135	116	141	141	115	169
7313	104	159	158	109	107	168	183	100	273	143	110	172	175	118	160	154	178	128	140	132	116	145	131	115	169
	104	159	167	109	111	172	198	100	281	147	110	172	178	118	160	162	186	128	140	132	116	147	131	115	169
12668	104	157	158	115	103	172	185	100	256	147	110	174	156	118	160	152	180	128	144	107	116	145	131	115	167
	104	161	172	117	103	172	188	100	259	151	110	182	169	118	160	154	184	128	160	132	116	145	131	115	169
5674	104	157	169	109	109	170	183	100	255	143	110	180	175	118	160	154	182	128	142	107	116	136	131	115	169
	106	161	169	115	109	170	187	100	261	153	110	189	175	118	160	154	182	128	144	129	116	136	131	115	169
GM288	104	157	167	109	107	167	183	100	273	143	110	172	175	118	160	154	188	128	140	132	116	145	131	115	169
	104	157	177	109	111	167	198	100	273	147	110	172	178	118	160	154	188	128	140	132	116	147	131	115	169
5355	104	159	167	109	107	172	183	100	261	147	110	172	175	118	160	154	188	128	130	132	130	145	131	115	169
	104	159	177	111	111	172	198	100	275	147	110	172	178	118	160	162	188	128	140	132	130	147	131	115	169
5565	104	159	169	109	109	170	183	100	255	141	110	180	175	118	160	156	182	128	142	107	116	136	131	115	169
	104	161	169	115	109	170	187	100	ND	153	110	182	175	118	160	156	182	128	144	129	126	136	131	115	169
T.SORD15	104	159	166	111	113	172	182	100	251	149	106	178	170	118	160	153	184	125	140	107	116	120	131	115	169
	104	163	166	111	113	172	182	100	251	149	106	178	170	118	160	153	196	128	142	132	116	120	131	115	169
MLD524	106	159	169	111	113	172	185	100	251	139	110	174	178	118	160	152	182	128	140	132	116	136	131	115	171

	106	161	167	111	115	172	196	100	259	147	110	174	178	118	160	152	186	128	140	135	116	136	131	115	171
9667	104	161	172	111	109	170	181	100	261	151	110	176	168	118	160	154	184	128	127	107	116	120	131	115	169
	104	161	172	111	109	170	181	100	261	151	110	176	168	118	160	154	184	128	127	132	116	122	131	115	169
12640	104	159	169	109	105	170	179	100	259	143	110	172	170	118	160	160	182	128	140	132	116	149	131	115	169
	104	161	167	115	105	170	183	100	259	145	110	172	170	118	160	162	188	128	140	138	116	154	131	115	169
JFV306	104	159	167	111	113	172	185	100	251	139	110	176	178	118	160	152	182	128	140	132	116	136	131	115	173
	106	163	169	111	115	172	196	100	259	147	110	176	178	118	160	162	186	128	140	135	116	136	131	115	173
JFV307	104	159	167	111	113	172	185	100	251	139	110	176	178	118	160	152	182	128	140	132	116	136	131	115	171
	106	161	169	111	115	172	196	100	259	147	110	176	178	118	160	162	186	128	140	135	116	136	131	115	171
5666	104	159	167	109	107	168	183	100	273	143	110	172	175	118	160	154	188	128	140	132	116	145	131	115	169
	104	159	177	111	111	172	198	100	275	147	110	172	178	118	160	162	188	128	140	132	116	147	131	115	169
5667	104	159	167	109	107	168	183	100	273	143	110	172	175	118	160	154	188	128	140	132	116	145	131	115	169
	104	159	177	111	111	172	198	100	275	147	110	172	178	118	160	160	188	128	140	132	116	147	131	115	169
6809	104	159	167	111	107	172	181	100	259	149	110	178	170	118	160	152	182	128	140	107	116	120	131	115	169
	104	163	172	111	109	172	187	100	ND	151	112	186	180	118	160	158	184	128	142	132	116	149	131	115	169
6824	104	163	172	111	109	172	181	100	259	149	110	178	170	118	160	154	184	128	140	107	116	122	131	115	169
	104	163	172	111	113	172	181	100	261	149	110	178	170	118	160	154	184	128	142	132	116	156	131	115	169
8622	104	159	167	111	107	172	181	100	259	149	110	178	170	118	160	152	182	128	140	107	116	120	131	115	169
	104	163	172	111	109	172	187	100	ND	151	112	186	180	118	160	152	184	128	142	138	116	149	131	115	169
9660	104	159	167	109	107	172	183	100	261	147	110	172	175	118	160	154	188	128	132	132	116	145	131	115	169
	104	159	177	111	111	172	183	100	275	149	110	172	178	118	160	154	188	128	140	132	116	147	131	115	169
11605	104	159	167	111	103	172	183	100	ND	141	106	180	168	118	160	154	184	128	132	132	108	154	131	115	169
	104	159	167	111	109	172	188	100	257	151	112	180	169	118	160	162	184	128	140	138	108	154	141	115	169
10171	104	163	166	111	109	172	181	100	259	149	110	182	170	118	160	154	184	125	140	107	116	152	131	115	169
	104	163	172	111	113	172	181	100	261	149	110	182	170	118	160	156	196	128	140	138	116	154	131	115	169
5340	104	159	167	109	107	172	183	100	261	147	110	172	175	118	160	154	188	128	132	132	116	145	131	115	169
	104	159	177	111	111	172	198	100	275	147	110	172	178	118	160	154	188	128	140	132	116	147	131	115	169
6716	104	163	167	111	109	172	183	100	253	139	110	170	168	118	160	152	182	128	142	107	116	136	131	115	169
	104	163	167	113	109	172	185	100	255	149	110	170	173	118	160	154	186	128	144	135	116	152	141	115	169
6723	104	159	169	113	109	172	183	100	257	143	110	170	169	118	160	152	182	128	140	107	116	136	141	112	169
	104	161	169	113	109	172	185	100	261	149	110	180	176	118	160	153	184	128	142	135	116	145	141	112	169
LBT964	104	159	167	111	103	172	179	100	255	147	110	170	168	118	160	150	182	128	130	107	116	154	131	115	169
	104	164	167	119	109	172	188	100	257	153	112	180	170	118	160	156	184	128	140	138	136	156	141	115	169
11604	104	159	167	111	103	172	183	100	257	141	110	182	169	118	160	154	184	128	130	138	116	154	131	112	169
	104	159	167	119	107	172	188	100	259	141	110	182	169	118	160	154	188	128	130	138	116	156	131	115	169
FNS258	104	159	167	111	109	172	183	100	255	141	106	182	168	118	160	154	182	128	130	107	116	156	131	115	169
	104	164	167	111	109	172	183	100	259	149	112	182	168	118	160	162	184	128	138	138	116	156	131	152	169
LBT966	106	159	167	111	109	172	183	100	257	151	110	172	169	118	160	154	182	128	130	107	116	145	131	115	169
	108	161	172	119	111	172	194	100	259	151	110	172	169	118	160	160	184	128	130	138	116	145	131	115	169
C12	104	159	169	111	113	172	185	100	253	139	110	174	178	118	160	152	182	128	140	132	116	136	131	115	169

	106	159	167	111	115	172	196	100	259	147	110	174	178	118	160	152	186	128	140	135	116	136	131	115	171
12626	104	159	167	109	109	172	183	100	256	145	110	172	168	118	156	150	184	128	140	132	116	152	131	115	169
	106	159	169	111	109	172	183	100	256	149	112	180	170	118	160	154	184	128	146	138	116	156	131	115	169
12624	104	167	100	159	105	172	183	100	257	151	106	174	168	118	160	150	182	128	130	107	116	156	131	115	169
	106	167	111	163	109	172	194	100	259	151	112	180	168	129	160	154	188	128	146	138	116	156	131	115	169
12628	104	159	167	100	105	172	183	100	257	151	106	174	168	118	160	150	182	128	130	107	116	145	131	115	169
	106	163	167	111	109	172	194	100	259	151	112	180	168	118	160	154	188	128	144	138	116	156	131	115	169
11640	104	163	172	111	109	172	181	100	257	143	106	178	170	118	160	154	184	125	140	132	116	122	131	115	169
	104	163	172	111	109	172	181	100	259	145	110	182	176	118	160	156	196	128	142	132	116	154	131	115	169
11639	104	163	172	111	109	172	181	100	259	149	106	178	170	118	160	154	184	128	142	107	116	122	131	115	169
	104	163	172	111	113	172	181	100	259	149	106	178	170	118	160	162	184	128	142	132	116	154	131	115	169
11629	104	159	167	100	105	172	183	100	257	151	106	174	168	118	160	150	182	128	130	107	116	156	131	115	169
	106	163	167	111	109	172	194	100	259	151	112	180	168	118	160	154	188	128	146	138	116	156	131	115	169
JFV313	104	159	167	111	113	172	185	100	251	139	110	176	178	118	160	152	182	128	140	132	116	136	131	115	169
	106	163	169	111	115	172	196	100	259	147	110	176	178	118	160	160	186	128	140	135	116	136	131	115	173
6813	104	161	172	111	109	172	181	100	259	149	106	178	170	118	160	152	184	128	146	107	116	120	131	115	169
	104	161	172	111	109	172	181	100	259	149	110	178	170	118	160	154	184	128	146	132	116	154	131	115	169
9538	104	159	167	111	107	172	181	100	259	147	110	178	170	118	160	152	182	128	140	132	116	120	131	115	169
	104	163	172	111	109	172	187	100	ND	149	112	186	180	118	160	152	184	128	142	138	116	149	141	115	169
8648	104	163	172	111	109	172	181	100	251	151	106	176	170	118	160	154	182	125	140	107	116	152	131	115	169
	104	163	172	111	111	172	181	100	251	151	106	178	170	118	160	154	184	128	142	132	116	154	131	115	169
3510	104	163	172	111	109	172	181	100	251	143	110	178	170	119	160	152	184	128	142	107	116	120	131	115	169
	104	163	172	111	109	172	181	100	259	143	110	178	170	119	160	152	184	128	142	132	116	154	131	115	169
9529	104	159	167	111	107	172	181	100	259	147	110	178	170	118	160	152	182	128	140	107	116	120	131	115	169
	104	163	172	111	109	172	187	100	ND	149	112	186	180	118	160	152	184	128	142	138	116	149	131	115	169
4250	106	157	166	111	103	172	183	100	255	143	110	178	170	118	160	156	184	125	138	107	116	142	131	115	169
	106	163	169	111	105	172	183	100	259	147	110	178	170	118	160	160	188	128	142	135	116	142	131	115	169
M1	104	159	166	111	111	172	181	100	251	149	110	176	170	118	160	152	196	125	142	107	116	122	131	115	169
	104	163	166	111	113	172	187	100	259	149	110	178	170	118	160	156	196	128	142	138	116	154	131	115	169
C48	104	159	167	111	113	172	185	100	251	139	110	176	178	118	160	152	182	128	140	132	116	136	131	115	173
	106	163	169	111	115	172	196	100	259	147	110	176	178	118	160	152	186	128	140	135	116	136	131	115	173
MLD291	104	159	167	111	113	172	185	100	251	139	110	166	178	117	160	152	182	128	140	132	116	136	131	115	171
	106	161	169	111	115	172	196	100	259	147	110	166	178	129	160	152	186	128	140	135	116	136	131	115	171
G15	104	159	169	109	107	172	183	100	255	141	110	180	175	118	160	156	182	128	142	129	116	141	131	115	169
	106	161	169	113	109	172	187	100	259	151	110	180	175	118	160	158	182	128	144	135	116	141	141	115	169
10290	104	159	167	109	103	172	183	100	255	151	110	184	169	118	160	150	184	128	138	107	116	147	131	115	169
	104	164	172	109	109	172	183	100	255	151	110	184	176	118	160	154	190	128	144	132	116	147	131	115	169
Cigs18	106	152	167	109	101	170	183	100	257	135	110	174	159	118	158	152	184	125	140	132	116	136	131	115	169
	106	155	172	119	107	174	185	100	259	145	110	176	166	118	160	158	186	128	140	135	116	149	131	115	169
6737	104	164	167	113	109	170	183	100	253	147	110	172	169	117	160	154	186	128	142	107	116	147	141	112	169

	106	164	167	113	109	172	185	100	261	147	110	176	169	117	160	162	186	128	144	132	116	152	141	115	169
MLCD44	106	159	166	111	103	172	183	100	255	143	110	178	170	118	160	156	184	125	138	107	116	142	131	115	169
	106	163	169	111	105	172	183	100	259	147	110	178	170	117	160	160	188	128	142	135	116	142	131	115	169
G41	104	157	167	109	103	172	183	100	256	141	110	172	173	117	160	150	182	125	140	107	116	142	131	115	169
	106	164	167	111	105	172	190	100	259	151	110	184	178	117	160	156	184	128	140	132	116	149	131	115	169
C45	104	161	167	111	113	172	185	100	251	139	110	176	178	118	160	152	182	128	140	132	116	136	131	115	173
	106	159	169	111	115	172	196	100	259	147	110	176	178	118	160	152	186	128	140	135	116	136	131	115	173
12630	104	159	167	111	105	172	183	100	257	149	110	174	169	118	160	150	184	128	130	132	116	147	131	115	169
	106	161	167	111	109	172	194	100	257	149	112	178	169	118	160	154	184	128	140	138	116	154	141	115	169
11609	104	159	167	119	103	172	179	100	257	145	110	176	170	118	160	154	184	128	130	107	116	154	131	115	169
	104	159	167	119	105	172	183	100	257	151	110	184	170	118	160	154	184	128	130	138	116	156	131	115	169
12903	106	155	166	111	103	170	183	100	255	143	110	180	170	118	160	156	184	125	138	107	116	142	131	115	169
	106	161	169	111	105	170	183	100	259	147	110	180	170	118	160	160	188	128	142	135	116	142	131	115	169
10285	104	155	174	109	103	170	183	100	256	141	110	180	176	118	160	154	184	128	140	107	116	147	131	112	169
	104	164	174	115	109	170	188	100	257	151	110	182	178	118	160	154	188	128	144	132	116	149	131	115	169
10268	104	159	167	109	107	170	183	100	256	151	110	180	180	118	160	154	186	128	140	107	116	147	131	115	169
	104	159	169	115	109	170	188	100	259	151	110	184	180	118	160	154	188	128	144	132	116	154	131	115	169
12667	106	159	169	111	103	172	191	100	256	139	110	182	156	118	160	154	186	128	142	107	116	145	131	115	169
	106	159	172	117	109	172	191	100	259	151	110	180	168	118	160	154	188	128	154	138	116	152	141	115	169
MLD490	104	159	167	111	113	172	185	100	251	139	110	176	178	118	160	152	182	128	140	132	116	136	131	115	169
	106	161	169	111	115	172	196	100	259	147	110	176	178	118	160	152	186	128	140	135	116	136	131	115	171
LBT1813	104	157	167	115	103	172	190	100	255	145	110	182	168	118	160	156	182	128	140	132	116	149	131	115	169
	104	157	172	115	103	172	190	100	259	151	110	186	175	118	160	158	186	128	142	138	116	156	131	115	169
9148	104	161	172	109	107	170	181	100	251	137	106	170	166	118	160	152	192	128	138	141	116	154	131	115	163
	104	164	172	111	109	170	187	100	ND	143	110	176	166	118	160	156	192	128	142	144	116	154	131	115	169

ND=no data
