# Supplementary material for: Dissecting the phyloepidemiology of Trypanosoma cruzi I (TcI) in Brazil by the use of high resolution genetic markers
Source: PLoS Negl Trop Dis. 2018 May 21;12(5):e0006466. doi: 10.1371/journal.pntd.0006466 (PMC5983858; doi:10.1371/journal.pntd.0006466)
Supplement: S8 Table — (PDF) [file pntd.0006466.s028.pdf]

S8 Table. Population genetic parameters for *a posteriori* sylvatic populations of TcI in Brazil

Population*	G/N	PL	PA/L ± SE	Ar ± SE	D_{AS} ± SD	Ho	He	%PL He	% PL Hd	F_{IS}± SE
1	17/17	20	0.61 ± 0.17	2.68 ± 0.31	0.403 ± 0.138	0.34	0.41	5	35	0.224 ± 0.08
2	15/15	18	0.72 ± 0.28	2.14 ± 0.27	0.331 ± 0.172	0.34	0.32	33.3	11.1	0.043 ± 0.14
3	38/38	25	1.26 ± 0.20	3.46 ± 0.27	0.491 ± 0.092	0.42	0.52	4	48	0.201 ± 0.04
4	5/5	15	0.06 ± 0.067	1.76 ± 0.39	0.459 ± 0.079	0.42	0.31	0	6.7	-0.397 ± 0.36
5	17/17	17	0.27 ± 0.12	1.74 ± 0.17	0.378 ± 0.208	0.37	0.27	52.9	29.4	-0.207 ± 0.19

***Population designation based on *a posteriori* DAPC cluster assignment.**

N: number of isolates in population; G: number of multilocus genotypes (MLGs) per population based on microsatellite data of 25 loci analyzed; PL: number of polymorphic loci out of 25 loci analysed; PA/L: mean number of private alleles per locus ± SE, calculated in HP-Rare (Kalinowski, 2005); Ar: allelic richness as a mean over loci ± SE, calculated in FSTAT 2.9.3.2 (Goudet, 1995); D_{AS}: mean pairwise allele sharing ± SD, calculated in MICROSAT v1.5d (Minch, 1997); Ho: mean observed heterozygosity across all loci, calculated in Arlequin v3.11 (Excoffier, 2005); He: mean expected heterozygosity across all loci, calculated in Arlequin v3.11 (Excoffier, 2005); %PL He: proportion of polymorphic loci showing a significant excess in heterozygosity after a sequential Bonferroni correction (Rice, 1989), calculated in Arlequin v3.11 (Excoffier, 2005); %PL Hd: proportion of polymorphic loci showing a significant deficit in heterozygosity after a sequential Bonferroni correction (Rice, 1989), calculated in Arlequin v3.11 (Excoffier, 2005); F_{IS}: mean fixation index ± SE, calculated in FSTAT 2.9.3.2 (Goudet, 1995).
