# Supplementary material for: Dissecting the phyloepidemiology of Trypanosoma cruzi I (TcI) in Brazil by the use of high resolution genetic markers
Source: PLoS Negl Trop Dis. 2018 May 21;12(5):e0006466. doi: 10.1371/journal.pntd.0006466 (PMC5983858; doi:10.1371/journal.pntd.0006466)
Supplement: S9 Table — (PDF) [file pntd.0006466.s029.pdf]

S9 Table. F_{ST} values in a five- way comparison between *a posteriori* populations (P<0.001)

	Population 1	Population 2	Population 3	Population 4	Population 5
Population 1	*				
Population 2	0.332	*			
Population 3	0.153	0.177	*		
Population 4	0.317	0.456	0.225	*	
Population 5	0.388	0.422	0.289	0.508	*
