# Supplementary material for: Dissecting the phyloepidemiology of Trypanosoma cruzi I (TcI) in Brazil by the use of high resolution genetic markers
Source: PLoS Negl Trop Dis. 2018 May 21;12(5):e0006466. doi: 10.1371/journal.pntd.0006466 (PMC5983858; doi:10.1371/journal.pntd.0006466)
Supplement: S10 Table — (PDF) [file pntd.0006466.s030.pdf]

S10 Table. Pairwise F_{ST} values for microsatellite data grouped according to the parasites' hosts.

	D_albiventris	*D_aurita*	*D_marsupialis*	Leontopithecus	Monodelphis	*P_opossum*	*P_frenatus*	Chiroptera	Rhodnius	Gracilinanus	*R_rattus*
D_albiventris		0.31	0.18	0.27	0.29	0.26	0.39	0.35	0.24	0.14	0.19
D_aurita	0.31		0.14	0.22	0.11	0.33	0.37	0.33	0.20	0.11	0.36
D_marsupialis	0.18	0.14		0.13	0.15	0.17	0.20	0.15	0.10	0.10	0.18
Leontopithecus	0.27	0.22	0.13		0.22	0.30	0.20	0.11	0.20	0.19	0.25
Monodelphis	0.29	0.11	0.15	0.22		0.28	0.37	0.33	0.17	0.13	0.34
P_opossum	0.26	0.33	0.17	0.30	0.28		0.42	0.38	0.18	0.25	0.29
P_frenatus	0.39	0.37	0.20	0.20	0.37	0.42		0.01	0.29	0.31	0.40
Chiroptera	0.35	0.33	0.15	0.11	0.33	0.38	0.01		0.26	0.25	0.40
Rhodnius	0.24	0.20	0.10	0.20	0.17	0.18	0.29	0.26		0.18	0.25
Gracilinanus	0.14	0.11	0.10	0.19	0.13	0.25	0.31	0.25	0.18		0.25
R_rattus	0.19	0.36	0.18	0.25	0.34	0.29	0.40	0.40	0.25	0.25	
median	0.27	0.26	0.15	0.21	0.25	0.29	0.34	0.30	0.20	0.18	0.27
average	0.26	0.25	0.15	0.21	0.24	0.29	0.30	0.26	0.21	0.19	0.29
	D_albiventris	***D_aurita***	***D_marsupialis***	**Leontopithecus**	**Monodelphis**	***P_opossum***	***P_frenatus***	**Chiroptera**	**Rhodnius**	**Gracilinanus**	***R_rattus***
	Cerrado	Atlantic Forest	Atlantic Forest	Atlantic Forest	Pantanal	Amazon	Atlantic Forest	Cerrado	Atlantic Forest	Pantanal	Caatinga
	Caatinga		Amazon		Caatinga				Amazon	Cerrado	

FDR = 0,016

(yellow) * 0,016 < p < 0,05

(orange) ** 0,001 < p < 0,016

(grey) *** p < 0,001
