# Supplementary material for: Dissecting the phyloepidemiology of Trypanosoma cruzi I (TcI) in Brazil by the use of high resolution genetic markers
Source: PLoS Negl Trop Dis. 2018 May 21;12(5):e0006466. doi: 10.1371/journal.pntd.0006466 (PMC5983858; doi:10.1371/journal.pntd.0006466)
Supplement: S17 Fig — Comparison between (A) MLST and maxicircle trees (B). (PDF) [file pntd.0006466.s017.pdf]

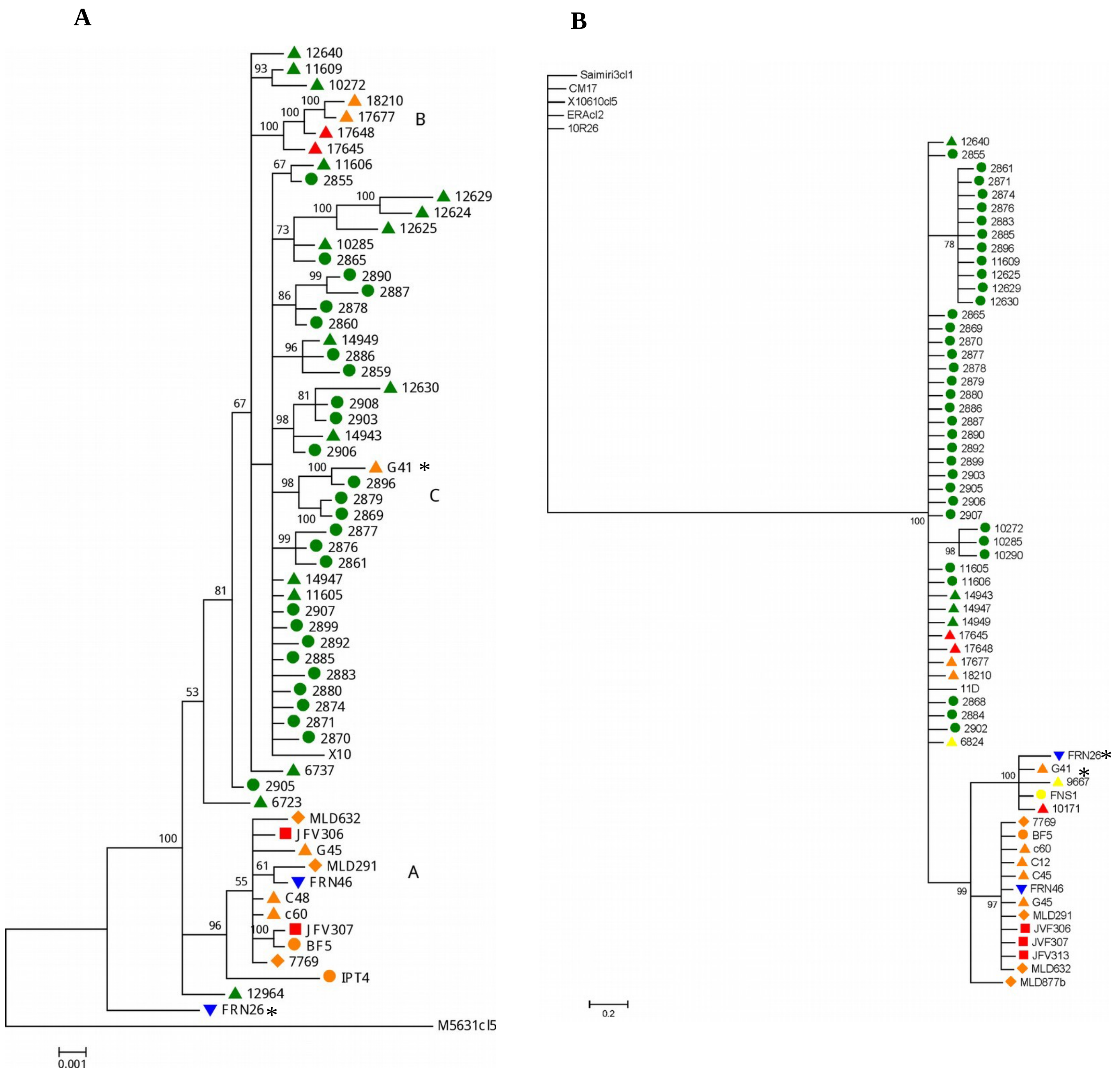

S17 Fig. Comparison between (A) MLST and maxicircle trees (B). Asterisk represent inter-lineage introgression events.
